# Evaluation of statistical methods for normalization and differential expression in mRNA-Seq experiments

**DOI:** 10.1186/1471-2105-11-94

**Published:** 2010-02-18

**Authors:** James H Bullard, Elizabeth Purdom, Kasper D Hansen, Sandrine Dudoit

**Affiliations:** 1 Division of Biostatistics, University of California, Berkeley, Berkeley, CA, USA; 2 Department of Statistics, University of California, Berkeley, Berkeley, CA, USA

## Abstract

**Background:**

High-throughput sequencing technologies, such as the Illumina Genome Analyzer, are powerful new tools for investigating a wide range of biological and medical questions. Statistical and computational methods are key for drawing meaningful and accurate conclusions from the massive and complex datasets generated by the sequencers. We provide a detailed evaluation of statistical methods for normalization and differential expression (DE) analysis of Illumina transcriptome sequencing (mRNA-Seq) data.

**Results:**

We compare statistical methods for detecting genes that are significantly DE between two types of biological samples and find that there are substantial differences in how the test statistics handle low-count genes. We evaluate how DE results are affected by features of the sequencing platform, such as, varying gene lengths, base-calling calibration method (with and without phi X control lane), and flow-cell/library preparation effects. We investigate the impact of the read count normalization method on DE results and show that the standard approach of scaling by total lane counts (e.g., RPKM) can bias estimates of DE. We propose more general quantile-based normalization procedures and demonstrate an improvement in DE detection.

**Conclusions:**

Our results have significant practical and methodological implications for the design and analysis of mRNA-Seq experiments. They highlight the importance of appropriate statistical methods for normalization and DE inference, to account for features of the sequencing platform that could impact the accuracy of results. They also reveal the need for further research in the development of statistical and computational methods for mRNA-Seq.

## Background

For the past decade, microarrays have been the assays of choice for high-throughput studies of gene expression. Recent improvements in the efficiency, quality, and cost of genome-wide sequencing have prompted biologists to rapidly abandon microarrays in favor of ultra high-throughput sequencing, a.k.a., second-generation or next-generation sequencing: e.g., Applied Biosystems' SOLiD, Helicos BioSciences' HeliScope, Illumina's Genome Analyzer, and Roche's 454 Life Sciences sequencing systems. These high-throughput sequencing technologies have already been applied to monitor genome-wide transcription levels (mRNA-Seq), DNA-protein interactions (ChIP-Seq), chromatin structure, and DNA methylation [1-9].

We evaluate statistical methods for the inference of differential expression (DE) with mRNA-Seq, using reference samples from the MicroArray Quality Control (MAQC) Project [10]. With corresponding quantitative real-time polymerase chain reaction (qRT-PCR) data on roughly one thousand genes, we compare different normalization and DE procedures and assess possible biases related to the sequencing technology. For genes that are well-expressed in both samples being compared, the examined tests (Fisher's exact test and GLM-based tests) are indistinguishable. However, substantial differences exist in their ability to give reliable DE estimates when even just one of the samples yields low read counts (e.g., ≤ 10). One inherent bias of the Illumina platform is the preferential sequencing of longer genes [11]. With the tests considered here, longer genes are more likely declared DE. We demonstrate that weighting the DE statistics by gene length can mitigate this effect.

While small "nuisance" technical effects can be observed due to differences in flow-cells/library preparations, we show that these do not impact substantially the differential expression calls for the MAQC dataset. We also find that not using the standard phi X control lane in each flow-cell, as in the base-calling calibration procedure recommended by Illumina, does not negatively impact DE detection. Moreover, auto-calibration without the phi X lane increases both quantity and quality of mapped reads. In this regard, there is no obvious benefit in using a phi X lane; doing away with such a control lane leads to more balanced and cost-effective designs.

We demonstrate that the greatest impact on DE detection is the choice of normalization procedure. As different lanes have different total read counts, i.e., *sequencing depths*, the usual approach is to scale gene counts within each lane by the total lane count: e.g., the now standard reads per kilobase of exon model per million mapped reads (RPKM) of [7] or the hypergeometric model of [6]. We show that this form of global normalization is heavily affected by a relatively small proportion of highly-expressed genes and, as such, can give biased estimates of DE if these few genes are differentially expressed across the conditions under comparison. We propose alternative more robust quantile-based normalization procedures that remove the bias without introducing additional noise.

## Methods

### MAQC datasets

This article considers two mRNA-Seq datasets related to the MicroArray Quality Control Project [10] and obtained using Illumina's Genome Analyzer II high-throughput sequencing system [12]. The experiments analyze two biological samples: Ambion's human brain reference RNA and Stratagene's human universal reference RNA, herein referred to as Brain and UHR, respectively.

In the first experiment (MAQC-2), two types of biological samples (Brain and UHR) were assayed, each using seven lanes distributed across two flow-cells. One library preparation was used for each of the two types of biological samples. Thus, biological effects are *confounded *with library preparation effects, i.e., some differences in mRNA-Seq measures between Brain and UHR could be due only to experimental artifacts. In the second experiment (MAQC-3), four different UHR library preparations were assayed using 14 lanes from two flow-cells; each library preparation was assayed on only one of the flow-cells. Thus, library preparation effects are *nested *within flow-cell effects and differences between flow-cells are confounded with library preparation effects (see [Additional file 1: Supplemental Figure S1] for the experimental design). Sequencing reads from both MAQC-2 and MAQC-3 experiments have been deposited to the short-read archive under the accession number, SRA010153.1.

As part of the original MAQC Project, around one thousand genes were also chosen to be assayed by qRT-PCR [13]. We use these qRT-PCR data as a gold-standard to benchmark the gene expression values determined by mRNA-Seq. Additionally, a large number of microarray experiments were conducted. We compare the mRNA-Seq measures to those derived from a set of Affymetrix Human Genome U133 Plus 2.0 arrays (GSE5350, samples AFX_1_ [A-B] [1-5]; see [Additional file 2: Supplemental Sections S1.2 and S1.2] for details on qRT-PCR and array analysis).

### Overview of the Illumina sequencing platform

We give a brief, non-technical overview of the steps involved in an Illumina mRNA-Seq experiment [12]. A sample of interest undergoes library preparation, a series of steps to convert the input RNA into small fragments of DNA that can be sequenced by the Illumina machine. Specifically, starting with any total RNA sample, Illumina's mRNA-Seq library preparation protocol includes poly-A RNA isolation, RNA fragmentation, reverse transcription to cDNA using random primers, adapter ligation, size-selection from a gel, and PCR enrichment [[14], Figure six]. The resulting cDNA *library *is placed in one of the eight *lanes *of a *flow-cell*. Individual cDNA fragments attach to the surface of the lane and subsequently undergo an amplification step, whereby they are converted into *clusters *of double-stranded DNA. The flow-cell is then placed in the sequencing machine, where each cluster is sequenced in parallel. Specifically, at each *cycle*, the four fluorescently labeled nucleotides are added and the signals emitted at each cluster recorded. For each flow-cell, this process is repeated for a given number of cycles, e.g., 35 cycles in the MAQC experiments. The fluorescence intensities are then converted into *base-calls*. The number of cycles determines the length of the *reads*; the number of clusters determines the number of reads.

### Pre-processing of sequencing data

For the two MAQC experiments, 35 base-pair-long reads were obtained using Illumina's standard Genome Analyzer pre-processing pipeline, Version 1.3 [12,15]. We used Bowtie to map reads to the genome (GRCh37 assembly) [16].

Illumina's default base-calling algorithm, Bustard, can be calibrated in two ways. The method recommended by Illumina is to reserve one lane per flow-cell for sequencing DNA (typically phi X DNA) and use data from this control lane to determine base-calls and quality scores for the other seven lanes [[15], Supplementary Information, p. 7]. Bustard can also be run using the auto-calibration method, which scores base-calls in a manner similar to the phred base-caller [17] and does not require a control lane per flow-cell. In both MAQC experiments, one lane of each flow-cell was reserved for sequencing phi X genomic DNA. For one experiment (MAQC-2), we obtained both auto-calibrated and phi X-calibrated reads.

Except for the section discussing the impact of base-calling calibration method, we focus on phi X-calibrated, purity-filtered reads that map uniquely to the genome, with up to two mismatches. The restriction to reads mapping to the genome implies that exon-exon junction reads are excluded (~10% of the reads). Additionally, the library preparation protocol does not allow consideration of strand-specific counts, so reads mapping to the forward and reverse strands are pooled.

### Definition of union-intersection genes

In our evaluation of DE, we focus on overall expression of a gene, rather than isoform-specific expression. There is no standard technique for summarizing expression levels of genes with several isoforms (see, for example, [6] and [7] for different approaches). For a given gene, we first define a *constitutive exon *as a set of consecutive exonic bases (i.e., portion of or entire exon) that belong to each isoform of the gene. We then define a *union-intersection (UI) gene *as a composite gene-level region of interest consisting of the union of constitutive exons that do not overlap with coding exons of other genes (based on Ensembl, Version 55; see [Additional file 2: Supplemental Section S2]). We retain all genes identified with chromosomes 1-22, X, and Y. In addition to including protein-coding genes, the UI genes represent a number of other classes of Ensembl annotation, such as pseudogenes and small RNAs.

### Normalization

In order to derive gene expression measures and compare these measures between (groups of) lanes, one first needs to normalize read counts to adjust for varying lane sequencing depths and potentially other technical effects. All but one of the normalization methods considered here are *global *procedures, in the sense that only a single factor *d*_*i *_is used to scale the counts (per-lane).

We evaluate three types of global normalizations: (1) total lane counts, as in RPKM of [7], (2) per-lane counts for a "housekeeping" gene expected to be constantly expressed across biological conditions, e.g., POLR2A, (3) per-lane upper-quartile of gene counts for genes with reads in at least one lane. In order to make the normalized expression measures comparable, the scaling factors are themselves scaled so that their sum across all lanes is equal to the sum of the total counts across all 14 lanes (see [Additional file 2: Supplemental Section S4]).

The expression quantitation problem can be framed in terms of generalized linear models (GLM),(1)

where the natural logarithm of the expected value of the read count *X*_*i, j *_for the *j*th gene in the *i*th lane is modeled as a linear function of the gene's expression level *λ*_*a*(*i*), *j *_for the biological condition *a*(*i*) assayed in lane *i *plus an offset (log *d*_*i*_) and possibly other technical effects (*θ*_*i, j*_).

Finally, we propose a quantile normalization procedure, inspired from the microarray normalization approach of [18] and its implementation in the R package aroma.light. Specifically, for each lane, the distribution of read counts is matched to a reference distribution defined in terms of median counts across sorted lane. The normalized data are rounded to produce integer values that can be used with the DE statistics described below.

### Differential expression

We compare three types of methods for inferring DE, each of which yields one test statistic per gene: Fisher's exact test statistic, likelihood ratio statistics based on a generalized linear model as in Equation (1), and *t*-statistics based on estimated parameters of the same GLM. Two different *t*-statistics are evaluated, which use different techniques for estimating the variance of the estimated parameters. We also assess the impact of flow-cell effects, either through the addition of parameters *θ*_*i, j *_in the GLM or through a Mantel-Haenszel test, an extension of Fisher's exact test (see [Additional file 2: Supplemental Section S5]). All of the considered DE statistics can accommodate global normalization via an offset *d*_*i*_. For the GLM-based statistics, the offset is handled as in Equation (1). Fisher's exact test and the Mantel-Haenszel test compare the distribution of the counts of the *j*th gene to that of *d*.

The likelihood ratio statistics are the most general, as they can be used for comparisons of any number of biological sample types and adjust for general experimental effects as well as sample covariates, e.g., RNA quality. The *t*-statistics are only applicable for testing differences between two groups. The *t*-statistics and likelihood ratio statistics are based on maximum likelihood estimators from the same GLM, but have different performance in certain cases. Distributional properties of all of the GLM-based statistics are derived under asymptotic theory; therefore, they may have poor behavior for small numbers of input samples or low counts (though this is not what we experience). In contrast, Fisher's exact test makes no assumption about sample size; however, it only adjusts for global experimental effects and even the Mantel-Haenszel extension allows only a single gene-level experimental effect.

Likelihood ratio statistics have been used in [6] for the special case of only a global lane effect (i.e., *θ*_*i, j *_= 0 in Equation (1)); these authors also mentioned applying an arcsine-root transformation for variance stabilization of the per-gene read proportions within each lane. Bayesian statistics with Gamma prior for the Poisson parameter have been found to yield similar results as the above GLM-based test statistics [19]. Other test statistics considered in the recent mRNA-Seq literature include *t*-statistics with square root-transformed standard errors and Bayesian statistics based on the Beta-Binomial distribution [3].

### Receiver operator characteristic curves using qRT-PCR gold-standard

The qRT-PCR data of [13] are used as gold-standard to determine "true" differential expression and derive receiver operator characteristic (ROC) curves for various mRNA-Seq and microarray DE methods. The qRT-PCR estimate of UHR to Brain expression log-fold-change is the difference of average expression measures for UHR and Brain across replicates (see [Additional file 2: Supplemental Section S6]).

We divide the genes assayed by qRT-PCR into three sets, "non-DE", "DE", and "no-call", based on whether their absolute expression log-fold-change is less than *a*, greater than *b*, or falls within the interval [*a*, *b*], respectively. We ignore the "no-call" genes when determining true/false positives/negatives. True positives (TP) are reported when the sequencing (or microarray) platform not only correctly declares a gene DE, but also agrees with qRT-PCR regarding the direction of DE. The true positive rate (TPR) is then defined as the total number of TPs divided by the total number of DE genes according to qRT-PCR; the false positive rate (FPR) is computed as usual. See Table 1 for a summary.

**Table 1:** Definition of true and false positive rates. Synopsis of the rules for calling true/false positives and negatives, which take into account the sign of the direction of differential expression: "+" for over-expression in UHR, "-" for over-expression in Brain.

		mRNA-Seq			
		Non-DE	DE +	DE-	
	Non-DE	TN	FP	FP	N
qRT-PCR	DE +	FN	TP	FP	P
	DE-	FN	FP	TP	

The true positive rate (TPR) is estimated as TP/P and the false positive rate (FPR) as FP/N.

## Software

In order to facilitate analysis and visualization of mRNA-Seq data, we developed two R/Bioconductor software packages, Genominator and GenomeGraphs [20]. Both packages are available from the Bioconductor Project, http://bioconductor.org/packages/release/bioc/html/Genominator.html and http://bioconductor.org/packages/release/bioc/html/GenomeGraphs.html, respectively.

## Results and Discussion

### Comparison of mRNA-Seq differential expression statistics

Lists of differentially expressed genes are typically produced by computing, for each gene, a test statistic comparing expression levels between the two types of biological samples and ranking the genes based on *p*-values assessing the statistical significance of the observed differences.

We evaluate various statistics for differential expression (see description in Methods, above) and find that the main difference between test statistics is their ability to handle low counts, an issue of great importance when investigating differential expression in context of mRNA-Seq. When both samples have zero reads, clearly nothing can be said about differential expression. The more pertinent zero-count or low-count scenario occurs when a gene has zero reads for one sample and a reasonable number for the other. Around 700 genes (~1.8%) have zero reads in either Brain or UHR and 10 or more reads in the other tissue. Presumably, this represents an interesting biological phenomenon, where a gene in one tissue is completely non-expressed according to sequencing.

**Figure 1:**
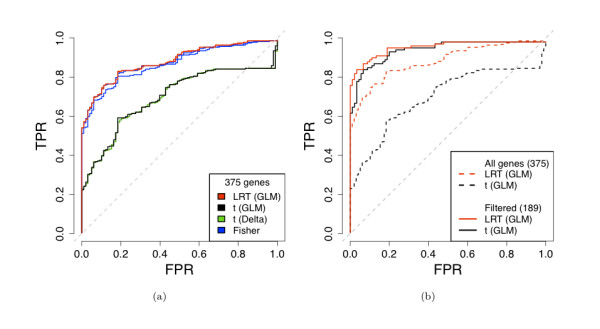
**Comparison of differential expression statistics: ROC curves**. (a) All DE statistics, no gene filtering. (b) GLM-based likelihood ratio statistics and *t*-statistics, before and after removing genes with fewer than 20 reads in either Brain or UHR. In both plots, a gene was declared non-DE if its qRT-PCR absolute log-ratio was less than 0.2 and DE if its absolute log-ratio was greater than 2.0. Note that we require a true positive to be differentially expressed in the same direction according to both mRNA-Seq and qRT-PCR (see Table 1 and Methods).

For genes with zero counts in either sample, the *t*-statistics fail: the estimated standard errors become extremely large (or infinite in the case of the delta method *t*-statistic) and the nominal *p*-values cluster around one, regardless of the number of reads in the other sample. For Fisher's exact test and the GLM-based likelihood ratio test, however, we see a continuum of *p*-values as desired. For genes with reasonable counts in both samples, the choice of test statistic makes little difference in the nominal *p*-values ([Additional file 1: Supplemental Figures S2 and S3]). Because they cannot stably handle low-count genes, the *t*-statistics are failing to detect many "easy" cases of DE (i.e., genes with large differences in expression between the two conditions) and, as a result, have very low sensitivity. The poor performance of the *t*-statistics is reflected in ROC curves (Figure 1). Removal of genes with fewer than 20 reads in either sample completely accounts for the poor sensitivity of the *t*-statistics and results in equivalent ROCs for the various DE statistics, all of which are dramatically improved (Figure 1).

As the different mRNA-Seq DE tests show similar behavior, we will from here on focus only on the results from the GLM-based likelihood ratio tests. The results do not change when different test statistics are used, except for the already noted poor performance of the *t*-statistics for low-count genes.

### Impact of technical effects on differential expression

#### Gene-length biases in differential expression

It is expected from the mRNA-Seq assay that longer transcripts contribute more "sequencible" fragments than shorter ones expressed at the same level. There is clearly a positive association between gene counts and length, an association that is not entirely removed via scaling by gene length, as in the RPKM of [7] ([Additional file 1: Supplemental Figure S4]). This suggests either higher expression among longer genes or non-linear dependence of gene counts on length.

**Figure 2:**
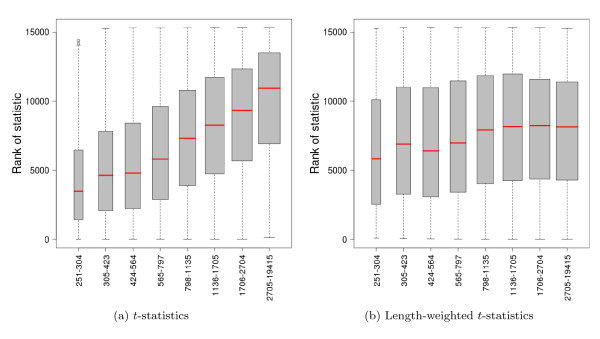
**Differential expression statistics, by length**. Boxplots of the ranks of DE statistics vs. gene lengths for UI genes at least 250 bp-long and with non-zero counts in both Brain and UHR. (a) Delta method *t*-statistics. (b) Delta method *t*-statistics weighted by the inverse of the square root of gene length.

As noted by [11], the dependence of gene counts on length creates "gene length-related biases" in mRNA-Seq DE results: longer genes tend to have more significant DE statistics (Figure 2). All of the mRNA-Seq DE statistics evaluated here have an inherent dependence of their estimated standard errors on read counts. This is a serious shortcoming in terms of creating "gene-lists" for differential expression, as the resulting lists could favor long genes with small underlying effects as compared to short genes with large effects. Considering only estimated fold-changes is inadequate, as this ignores the fairly large range of standard errors for a given fold-change and gene length.

One can possibly remedy the length dependence of DE statistics using a fixed number of bases from each gene; repeating the DE analysis by randomly selecting 250 bp from each gene removes the association between DE significance and length ([Additional file 1: Supplemental Figure S5]). This also indicates that the cause of the association is the length of the gene and not differences in the underlying expression levels of longer genes. However, a fixed-length analysis is unsatisfactory, as it discards large amounts of data and there is no natural choice of common length.

A weighted analysis based on gene length might constitute a reasonable compromise towards a length-independent DE filter. Indeed, scaling each *t*-statistic by the inverse of the square root of length provides a length-independent ranking (Figure 2). However, the problem of choosing a cutoff still remains. Under the assumptions presented in [11], with the unweighted *t*-statistics and using the same cutoff across genes, power increases with gene length for a given level of DE. Under the same scenario, for the weighted *t*-statistics, both Type I error rate and power decrease with length.

#### Impact of base-calling calibration method

**Figure 3:**
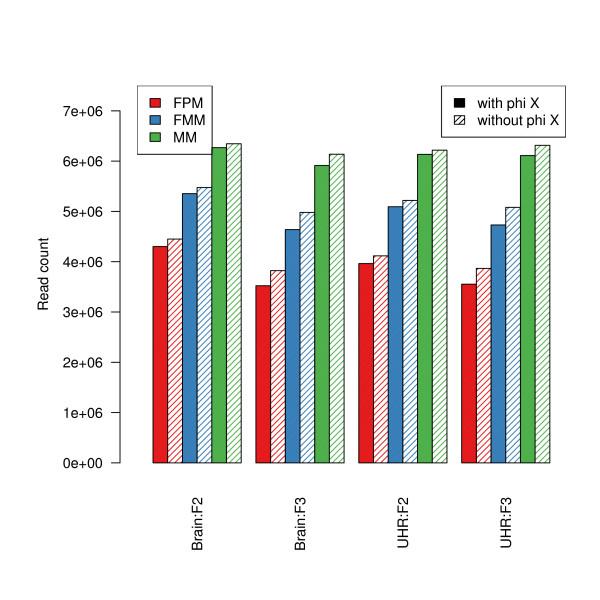
**Impact of base-calling calibration method on read-mapping**. Barplots of average read counts per lane with and without phi X calibration, for each of the four biological sample (Brain, UHR) and flow-cell (F2, F3) combinations. Reads are classified into three nested categories: purity-filtered perfectly matching reads (FPM); purity-filtered reads with either 0, 1, or 2 mismatches (FMM); unfiltered reads with either 0, 1, or 2 mismatches (MM).

The practice of reserving one lane out of eight, in each flow-cell, for sequencing bacteriophage phi X genomic DNA has important implications for experimental design, in terms of sample size and balance. We find that more reads are mapped to the genome with auto-calibration than with the standard phi X calibration, at each of three mapping stringency levels (Figure 3). Purity-filtered perfectly matching (FPM) reads are unlikely to contain sequencing errors and can serve as proxies for perfectly accurate reads. Similarly, purity-filtered reads with either 0, 1, or 2 mismatches (FMM) are comprised of both FPM reads as well as reads that represent sequencing errors. Then, the ratio (FMM-FPM)/FMM can be viewed as a rough estimate of the sequencing error rate, assuming no SNPs. For all lanes, the auto-calibration method produces slightly lower error rates (by ~5%).

The increased number of reads is spread unevenly throughout the transcriptome. A majority of the UI genes have no change in read counts between calibration methods, whereas around 25% of the genes have 4 or more additional reads when using auto-calibration. When computing an (FMM-FPM)/FMM ratio for each gene for both phi X and auto-calibration, auto-calibration yields lower error rates by about 3.8% on average.

The significance of differences in expression measures between the two calibration methods was evaluated by comparing observed differences to a permutation distribution of differences obtained by randomly swapping the auto-calibrated and phi X-calibrated sets of read counts for each of the 14 lanes. We find that in terms of absolute expression measures there are small, but statistically significant differences between the two calibration methods. However, relative expression measures, as used in DE analyses, do not appear to be significantly different (see [Additional file 2: Supplemental Section S8]).

Although our assessment is based on only two flow-cells, it seems quite clear that auto-calibration is advantageous, as it yields more balanced designs, frees up one lane per flow-cell, and produces a larger number of higher quality reads per lane.

#### Lane, flow-cell, and library preparation effects

The Poisson distribution has been shown to provide a good fit to the distribution of gene-level counts across replicate lanes, after normalization by total lane counts [4,6]; our experience with both the MAQC data and unpublished datasets for *Drosophila melanogaster *supports this conclusion. The goodness-of-fit of the Poisson model across different organisms and different sequencing facilities strongly supports its validity as a model for lane variation and justifies the pooling of read counts across lanes by summation. Note, however, that the applicability of the Poisson distribution is questionable when analyzing *biological replicates *(i.e., samples from different individuals within a given biological group, such as, patients with the same type of cancer). The use of negative binomial or empirical Bayes methods, as described in the SAGE literature [21,22], may be sensible in such settings of increased variability.

Our analyses also confirm the previously noted small technical differences between flow-cells [6], though there is evidence of slightly more variation between flow-cells than between replicate lanes ([Additional file 1: Supplemental Figure S6c]). Regardless of their statistical significance, estimated flow-cell effects are small in magnitude and thus have a minor impact only in detecting extremely small biological effects; almost none for genes with more than 3 reads/lane.

**Figure 4:**
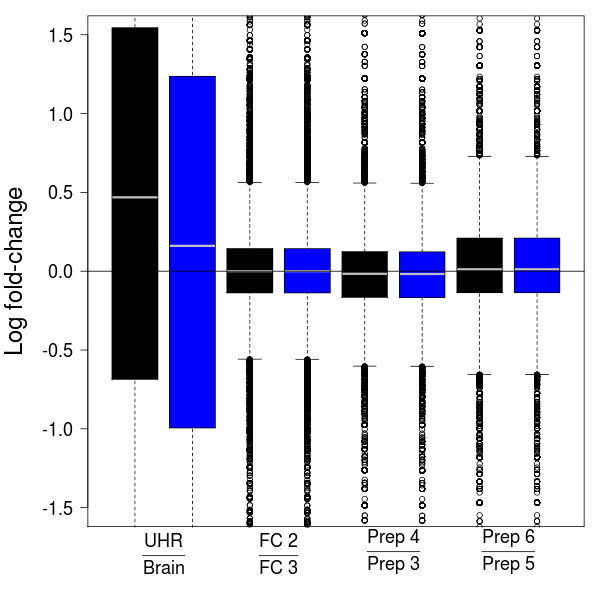
**Comparison of biological, library preparation, and flow-cell effects**. Boxplots of estimated log-fold-changes for UHR vs. Brain biological effects (GLM 2 in [Additional file 2: Supplemental Table S4]), flow-cell effects adjusting for biology (GLM 4), library preparation effects within flow-cell (GLM 7). Estimates are presented for total-count (black) and upper-quartile (blue) normalization.

To the best of our knowledge, there has been no published examination of the technical variation introduced during library preparation; replication of the library preparation is both expensive and time-consuming. There are clear library preparation effects on the total number of reads ([Additional file 1: Supplemental Figure S1]). After adjusting for differences in total lane counts, there is evidence for increased variation across replicate library preparations as compared to flow-cells and lanes ([Additional file 1: Supplemental Figure S6d]); however, this increased variability is mainly due to high-count genes for which there is high power to detect small differences. A direct comparison of library preparation effects to flow-cell and biological effects is not possible due to the experimental design, but comparison of the magnitude of the estimated differences suggests that library preparation effects are much smaller than the biological effects between Brain and UHR (Figure 4) and slightly larger for some genes than flow-cell effects (Figures 4 and [Additional file 1: Supplemental Figure S6]).

The biological differences between Brain and UHR samples may be much larger than those typically observed; therefore, technical sources of variation need not always be irrelevant. Finally, we note that the MAQC data are somewhat "ideal", in the sense that: (1) commercial-grade RNA was sequenced and (2) the sequencing was performed in-house by Illumina. A typical mRNA-Seq experiment begins with the extraction of RNA from biological specimens and variability induced during extraction may be much larger than the technical variability seen here.

### Normalization of mRNA-Seq data

Because the total number of reads varies between lanes, read counts must be normalized to allow comparison of expression measures across lanes or samples. While this subject has received relatively little attention in the mRNA-Seq literature, the common practice is to scale the gene counts by lane totals [6,7]. We find, however, that more general quantile-based procedures yield much better concordance with qRT-PCR and are hopefully more robust than normalization by a single housekeeping gene.

Here, we evaluate a variety of normalization procedures and focus on two main questions: (1) Does the normalization improve DE detection (sensitivity)? (2) Does the normalization result in low technical variability across replicates (specificity)? To assess DE detection, we rely on the qRT-PCR data of [13] as a gold-standard for determining true and false positives. Because there are a limited number of non-DE genes in the qRT-PCR data, we also assess goodness-of-fit to the Poisson model for replicate lanes (GLM 1 in [Additional file 2: Supplemental Table S4]).

The simplest form of normalization is achieved by scaling gene counts, in lane *i*, by a single lane-specific factor *d*_*i*_. In essence, these *global *scaling factors define the null hypothesis of no differential expression: if a gene has the same proportions of counts across lanes as the proportions determined by the vector of *d*_*i*_'s, then it is deemed non-differentially expressed.

**Figure 5:**
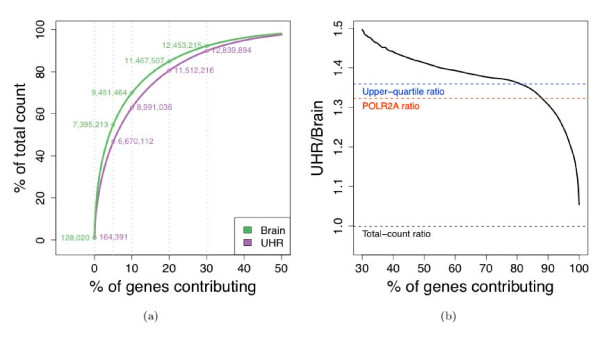
**Impact of highly-expressed genes**. (a) Cumulative percentage of total read count for Brain (green) and UHR (purple) samples, starting with the gene with the *highest *read count (across the seven Brain or UHR lanes). Cumulative read counts are marked for the 5, 10, 20, and 30 percent most highly expressed genes. (b) Running value of the UHR/Brain expression fold-change for unnormalized counts, starting with the gene with the *lowest *total count across all 14 lanes. Horizontal lines correspond to: the ratio of the counts for all genes (black), the ratio of the counts for the POLR2A gene (red), and the ratio of the per-lane upper-quartile of counts for genes with reads in at least one lane (blue).

The standard total-count normalization results in low variation across lanes, flow-cells, and library preparations, as discussed above. What has not been understood previously, is that this normalization technique reflects the behavior of a relatively small number of high-count genes: 5% of the genes account for approximately 50% of the total counts in both Brain and UHR. These genes are not guaranteed to have similar levels of expression across different biological conditions and, in the case of the MAQC-2 dataset, they are noticeably over-expressed in Brain, as compared to the majority of the genes (Figure 5).

**Figure 6:**
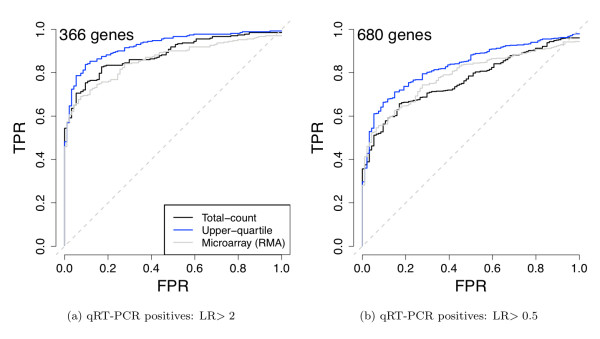
**Comparison of mRNA-Seq and microarray differential expression calls to qRT-PCR: ROC curves**. Genes common to all three platforms and present for both qRT-PCR and sequencing (see [Additional file 2: Supplemental Section S6]). evaluated and declared DE if their qRT-PCR absolute log-ratio was (a) greater than 2 or (b) greater than 0.5; genes were declared non-DE if their absolute log-ratio was less than 0.2. The GLM-based likelihood ratio test was used for the sequencing data. Two normalization procedures are presented for mRNA-Seq: total-count (black) and upper-quartile (blue) normalization. Microarray data were normalized using RMA (gray). Note that we require a true positive to be differentially expressed in the same direction according to both mRNA-Seq and qRT-PCR (see Table 1 and Methods).

Accordingly, the performance of total-count normalization is not particularly impressive for detecting DE (Figure 6): sensitivity is only slightly higher as compared to the microarray data, even for genes with relatively large differences in expression (> 2 absolute log-ratio). When including genes with lower levels of differential expression (> 0.5 absolute log-ratio), performance is no better (and perhaps slightly worse) than that of microarrays. This contradicts general expectation given that the mRNA-Seq data are less noisy and thus better at detecting small expression differences. For small levels of DE, the bias in estimated log-ratios using total-count normalization makes the sequencing estimates less accurate. 

We evaluate two alternatives for normalization of mRNA-Seq data. One approach relies on a single housekeeping gene like POLR2A, a standard technique for normalizing qRT-PCR expression measures. However, this is not a feasible solution in general, since it is not known *a priori *which genes have stable expression levels (in [13], POLR2A was chosen only after examining many replicates for UHR and Brain across a number of plates).

In analogy with standard techniques for normalizing microarray data, we propose to match the between-lane distributions of gene counts in terms of parameters such as quantiles. For instance, one could simply scale counts within lanes by their median. In our case, due to the preponderance of zero and low-count genes, the median is uninformative for the different levels of sequencing effort. Instead, we use the per-lane upper-quartile (75th percentile), after excluding genes with zero reads across all lanes (see Methods).

**Figure 7:**
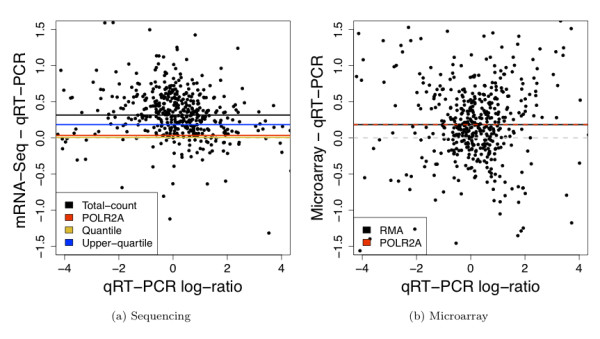
**Comparison of mRNA-Seq and microarray differential expression measures to qRT-PCR**. Difference scatterplots comparing the estimates of UHR/Brain expression log-ratio from qRT-PCR to those from (a) mRNA-Seq, using the standard total-count normalization, and (b) microarrays, using the standard RMA normalization. Shown are the genes shared between all three platforms, present in both Brain and UHR according to both mRNA-Seq and qRT-PCR (see [Additional file 2: Supplemental Section S1]), and having absolute qRT-PCR expression log-ratio less than 4. Horizontal lines in (a) represent the median UHR/Brain log-ratio for the sequencing data after the standard total-count normalization (black), POLR2A normalization (red), quantile normalization (yellow), upper-quartile normalization (blue); horizontal lines in (b) show the median UHR/Brain log-ratio for the microarray data after the standard RMA normalization (black) and POLR2A normalization (red).

Compared to total-count normalization, both POLR2A and upper-quartile normalization significantly reduce the bias of DE relative to qRT-PCR (Figure 7 and [Additional file 1: Supplemental Figure S7]), with upper-quartile having bias near zero. ROC curves illustrate that both upper-quartile and POLR2A normalization are unequivocally better than total-count normalization at detecting DE (Figure 6 and [Additional file 1: Supplemental Figure S8a]) and result in improved sensitivity of sequencing relative to microarray data (Figure 6 and [Additional file 1: Supplemental Figure S9]).

**Figure 8:**
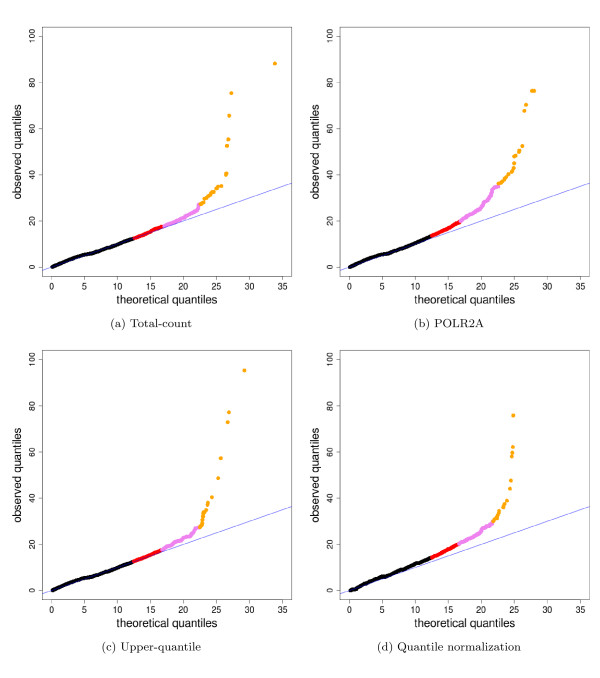
**Comparison of normalization procedures: Goodness-of-fit of Poisson model**. The multiplicative Poisson model (GLM 1 in [Additional file 2: Supplemental Table S4]) is fit to the seven Brain lanes in the MAQC-2 experiment after (a) total-count, (b) POLR2A, (c) upper-quartile, and (d) quantile normalization. Goodness-of-fit statistics are computed and displayed in *χ*^2 ^quantile-quantile plots. Genes with goodness-of-fit statistics in the top quantiles of the *χ*^2^-distribution are displayed using colored plotting symbols: red (1, 5]%, purple (.1, 1]%, gold [0, .1]%. Similar plots for UHR show the same patterns.

A closer look at technical variation for the different normalization procedures shows that upper-quartile normalization does not noticeably increase the level of variability as compared to total-count normalization; POLR2A normalization is slightly more variable but still comparable (Figure 8). 

Finally, it is also feasible to perform quantile normalization across lanes, as is often done in microarray experiments [23]. However, there does not seem to be added benefit to this more complicated normalization strategy. Quantile normalization performs similarly in the ROC analyses (Figure [Additional file 1: Supplemental Figure S8a]) and induces comparable, or even slightly more, variability than upper-quartile normalization (Figure 8). We again recall the somewhat artificial nature of the MAQC data, which were obtained at essentially the same time, by one lab, using ideal RNA samples. As more data become available, there may be larger variations in gene count distributions necessitating more aggressive normalization.

## Conclusions

Our main novel finding is the extent to which normalization affects differential expression results: sensitivity varies more between normalization procedures, than between test statistics. Although the standard total-count normalization results in Poisson variation across replicate lanes, it has poor detection sensitivity when benchmarked against qRT-PCR. Instead, we propose scaling gene counts by a quantile of the gene-count distribution (the upper-quartile) and show that such normalization improves sensitivity without loss of specificity.

An important aspect of the MAQC datasets, which could have an impact on the interpretation of the analyses presented, is the large difference in gene expression between Brain and UHR. Often, gene expression analyses consider much more closely related sets of samples, with only relatively few genes expected to be differentially expressed. In the comparison of Brain and UHR, by contrast, only 5-30% of genes examined by qRT-PCR were deemed as *non*-differentially expressed (depending on the choice of the multiple testing procedure used to correct the *p*-values). Indeed, there may be no truly non-DE genes queried by the qRT-PCR experiment, but rather, very small differences in expression for every gene. This creates a possibility for errors when specifying a set of true negatives; we have tried to control for this by a careful and stringent definition of true negatives and by evaluating the effect of changes in this definition (see [Additional file 2: Supplemental Section S6]).

Furthermore, the extreme difference in transcriptional profiles between the Brain and UHR samples means that the *p*-values from the sequencing experiment are smaller than would be expected if all the genes were truly non-DE. In particular, the *p*-values for non-DE genes (according to qRT-PCR) do not follow the expected uniform distribution, but are noticeably shifted toward zero ([Additional file 1: Supplemental Figure S10]). The microarray data demonstrate the same behavior ([Additional file 1: Supplemental Figure S10]), suggesting it is caused by the samples under consideration and not by inherent problems of the statistical methods. In contrast to the qRT-PCR tests for differential expression, the tests applied to sequencing data take into account the total number of reads mapping to each gene and, as a result, tend to have greater power for longer genes.

Another possible critique is that the improvement of UQ over total-count normalization is due to this normalizations more closely matching the *normalization *procedure used with the qRT-PCR data rather than proper reflection of actual biological differences; in other words, UQ normalization might be closely matching the effect of dividing by POLR2A, as is done with the qRT-PCR data but not the underlying biology. Indeed, additional scaling of the microarray data by POLR2A slightly improves the ROC compared to the standard microarray quantile normalization ([Additional file 1: Supplemental Figure S8b]). It is more likely, however, that total-count normalization, with its reliance on high-count genes, poorly reflects biological differences. This can be seen by taking a closer look at the POLR2A gene, which was chosen as a reference for qRT-PCR data because of its very similar expression in UHR and Brain across many qRT-PCR replicates [13]: the UHR to Brain fold-change of POLR2A is estimated as 1.3 for total-count normalization in contrast to 0.97 for upper-quartile normalization and 0.90 for microarray data. 

In regards to DE test statistics, the GLM-based likelihood ratio statistics and Fisher's exact statistics perform equally well in terms of sensitivity and handling of low-count genes. We find likelihood ratio tests appealing because of their generality. Indeed, using the GLM framework, one can adjust for potential confounding variables, including quantitative covariates, e.g., age of sample, as well as accommodate different count distributions (negative binomial in cases of over-dispersion).

A serious concern with all the DE methods considered here is the inherent dependence of power on read count, which in turn is related to both gene expression level and length. As most DE studies produce gene-lists, which are often then related to functional annotation (e.g., GO), it is undesirable for significance values to be driven by features such as length. A weighted analysis based on gene length might lead to a reasonable length-independent ranking of genes, that would allow short genes with large effects to gain in significance compared to long genes with small effects.

We find that technical variation is quite low across lanes and flow-cells and slightly larger across library preparations. In all cases, however, the effect on differential expression results is minimal. As noted above, the MAQC datasets are unusual, in that we expect extremely large differences in expression between Brain and UHR and only small library preparation effects because of the high quality of the RNA. In practice, library preparation effects may be closer in magnitude to biological effects.

We have demonstrated that while there are some differences between phi X and auto-calibration in the early stages of the analysis pipeline, the differences in terms of differential expression are small. Overall, auto-calibration seems advantageous, as it yields more balanced designs, frees up one lane per flow-cell, and produces a larger number of higher quality reads per lane.

The analysis conducted in this work, as well as others, is predicated on a "whole-gene" view of expression profiling. We evaluated technical effects, phi X calibration, and normalization methods using a very constrained UI gene definition. We limited ourselves to such a strict definition in order to ensure that the evaluation was not biased by alternative splicing or overlapping genes. Our UI gene definition is a gross over-simplification, as a large amount of biologically relevant information is lost; we exclude more than 50% of the reads which fall within Ensembl genes.

As high-throughput sequencing becomes more prevalent, our ability to precisely characterize the transcriptome of a sample will dramatically increase. More refined analyses, such as isoform-level expression, allele-specific expression, and genome annotation (segmentation), involve comparing distinct regions within a sample as opposed to the same region across samples. Such analyses will require an understanding of the effect of sequence composition on base coverage to account for the heterogeneity of base-level count distributions

## Authors' contributions

JB processed the data, co-wrote the Genominator package, conducted statistical analyses, and drafted the manuscript. EP conducted statistical analyses and drafted the manuscript. KH co-wrote the Genominator package and assisted in drafting earlier versions of the manuscript. SD drafted the manuscript and designed and coordinated the research. All authors read and approved the final manuscript.

## Supplementary Material

Additional file 1 **Supplementary Figures File**. Additional figures referred to in the main article. Click here for file

Additional file 2 **Supplementary Text File**. Additional text to describe further details and results of the analysis. Click here for file
