# Supplementary material for: Evaluation of statistical methods for normalization and differential expression in mRNA-Seq experiments
Source: BMC Bioinformatics. 2010 Feb 18;11:94. doi: 10.1186/1471-2105-11-94 (PMC2838869; doi:10.1186/1471-2105-11-94)
Supplement: Additional file 1 — Supplementary Figures File. Additional figures referred to in the main article. [file 1471-2105-11-94-S1.PDF]

## Supplementary Figures

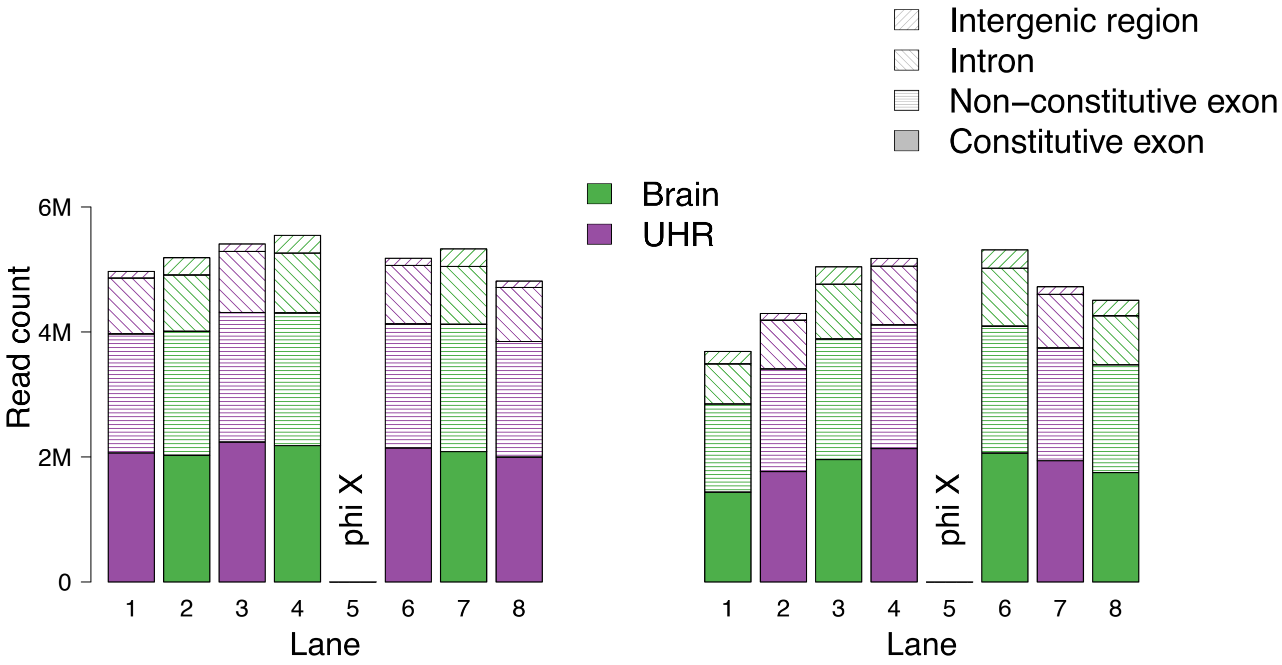


(a) MAQC-2

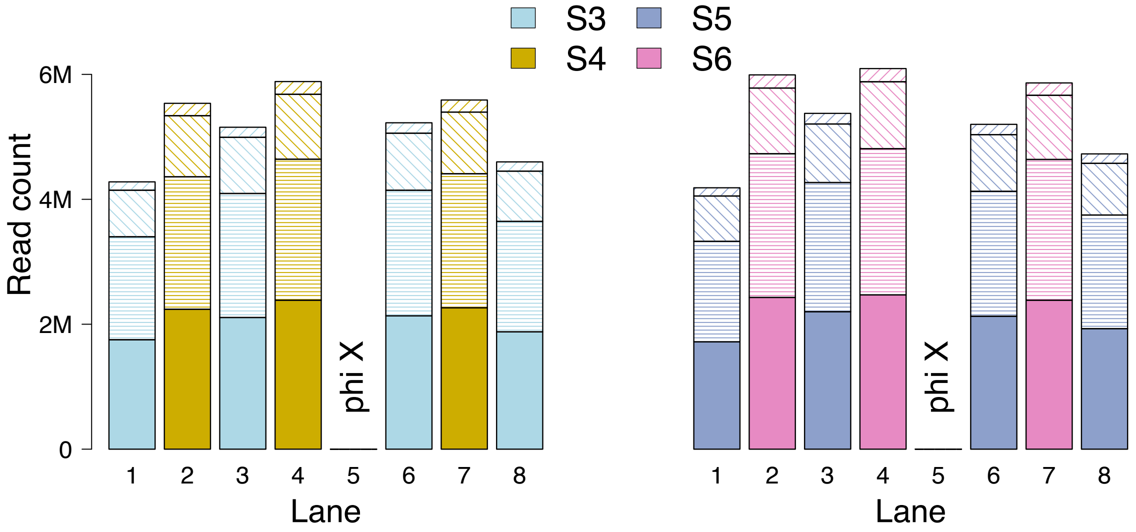


(b) MAQC-3

Figure S1: *Experimental design and per-lane read counts.* Barplots of per-lane read counts for: (a) MAQC-2 experiment and (b) MAQC-3 experiment. There are fourteen lanes in each experiment. The MAQC-2 experiment assayed single library preparations of Brain and UHR RNA, each using seven lanes distributed across two flow-cells. The MAQC-3 experiment assayed four different library preparations of UHR RNA, each across 3-4 lanes within a single flow-cell. The fifth lane in each flow-cell was reserved for phi X genomic DNA. Only purity-filtered reads that map uniquely to the genome with up to two mismatches in the first 35 bases (FMM) are retained. Total lane counts are partitioned into read counts for introns, constitutive exons, non-constitutive exons, and intergenic regions.

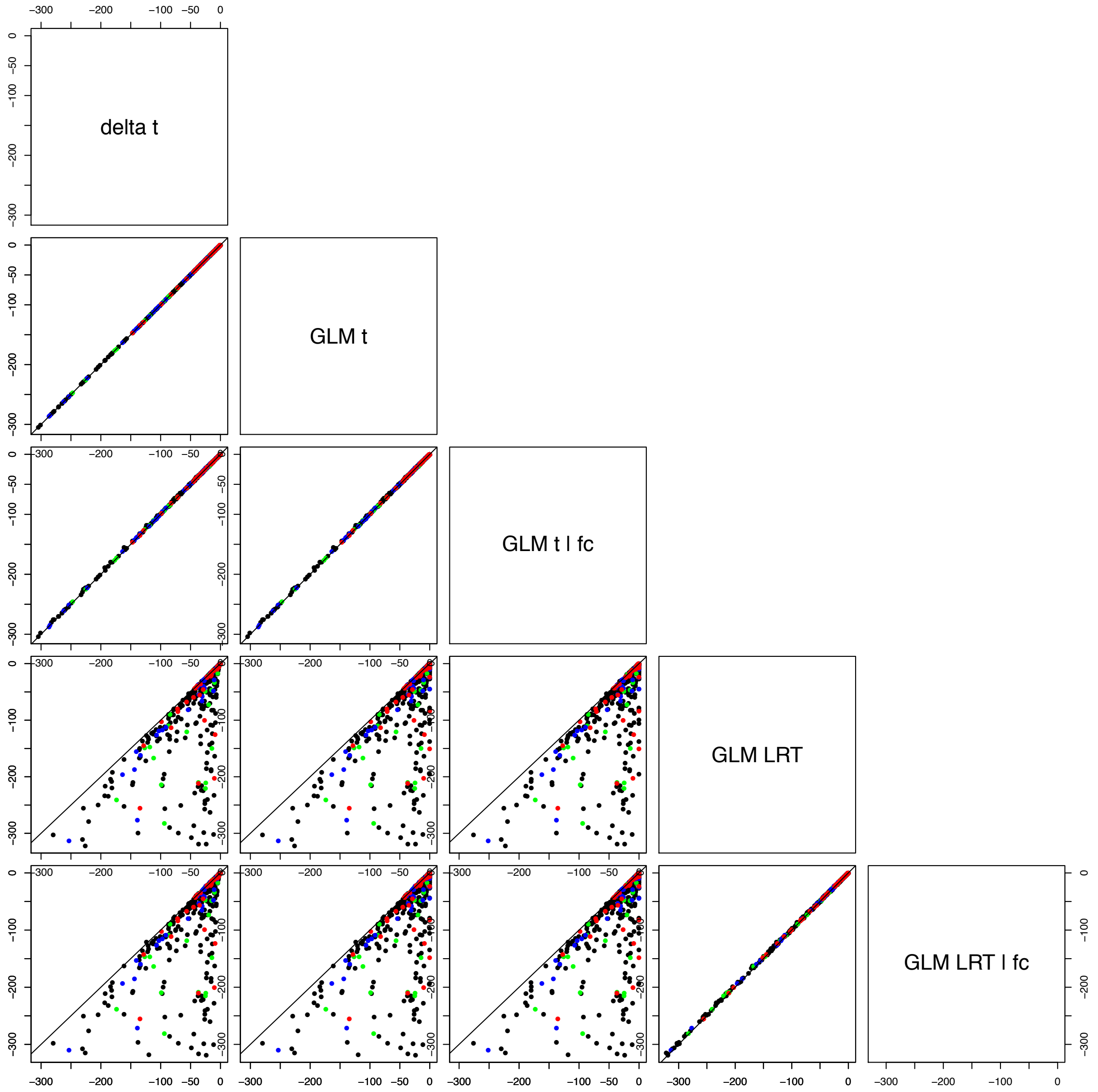


Figure S2: *Comparison of GLM-based DE statistics.* Scatterplot matrix of nominal $p$-values on the log-scale for differential expression statistics for genes assayed by both mRNA-Seq and qRT-PCR: $t$-statistics with standard errors calculated by the delta method (delta t), standard GLM $t$-statistics with and without flow-cell effects (GLM t | fc and GLM t), and GLM likelihood ratio statistics with and without flow-cell effects (GLM LRT | fc and GLM LRT). Genes with zero $p$-values are not displayed. Plotting symbols are colored according to the read counts of the corresponding gene in the Brain and UHR samples. **Black**: $\geq 6$ reads for both Brain and UHR; **Green**: $\geq 6$ reads for Brain, $< 6$ reads for UHR; **Blue**: $< 6$ reads for Brain, $\geq 6$ reads for UHR; **Red**:$< 6$ reads for both Brain and UHR.

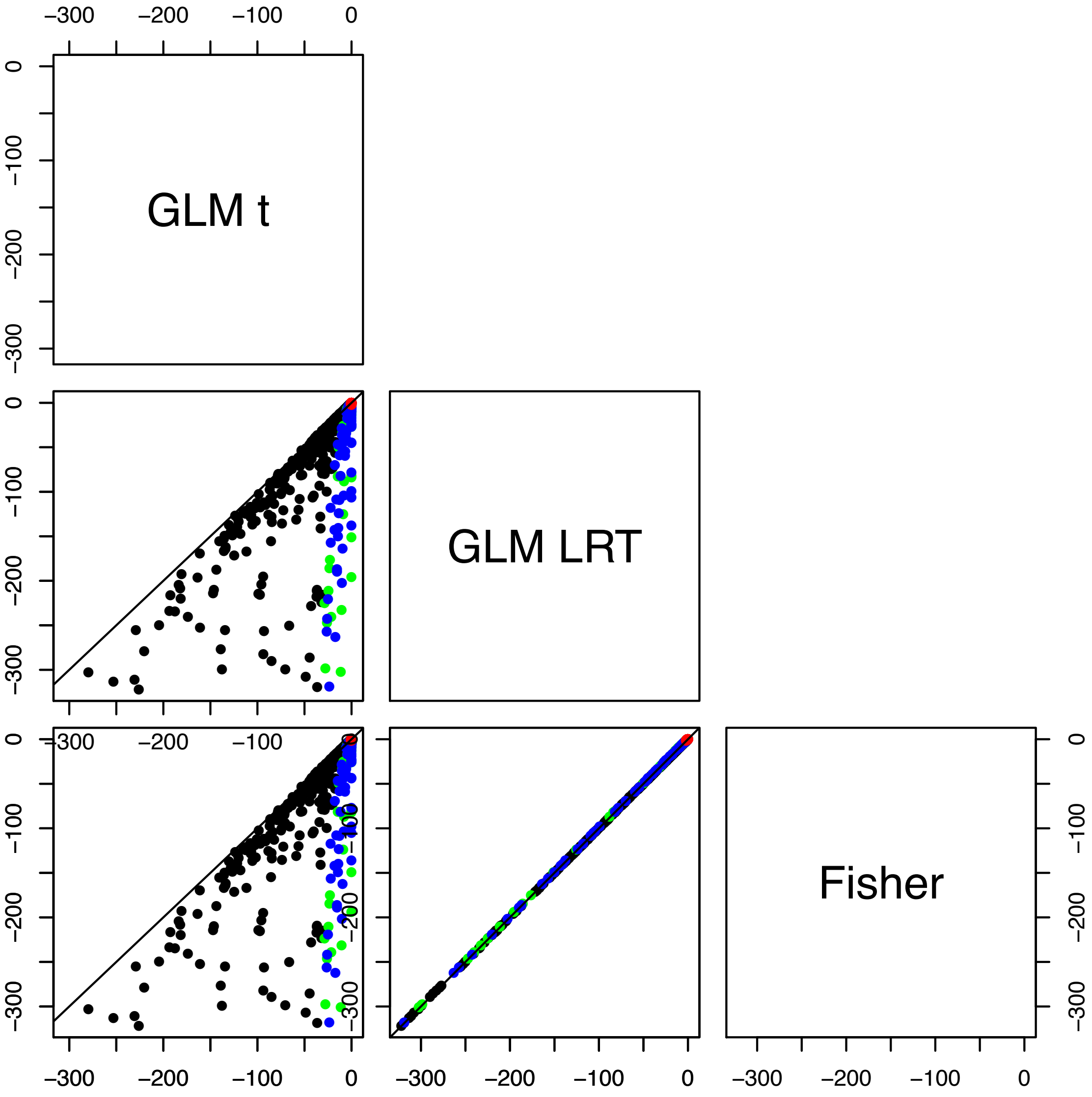


Figure S3: *Comparison of GLM-based and Fisher DE statistics.* Scatterplot matrix of nominal $p$-values on the log-scale for differential expression statistics for genes assayed by both mRNA-Seq and qRT-PCR. Plotting symbols are as described in the caption of Figure S2.

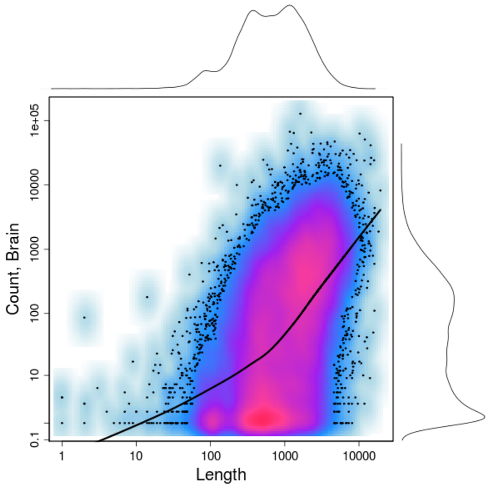


(a) Count vs. length

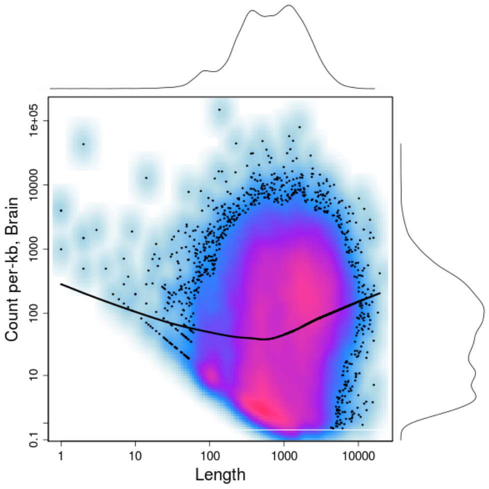


(b) Count per-kb vs. length

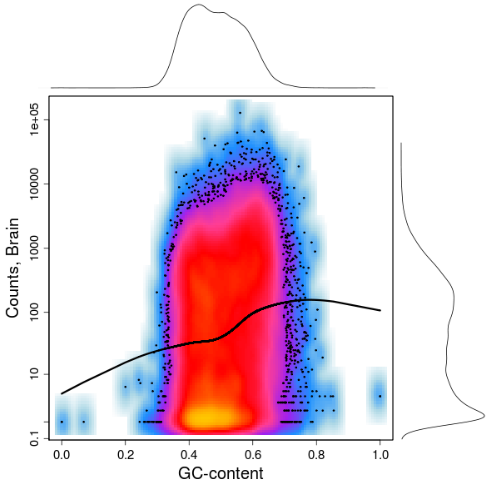


(c) Count vs. GC-content

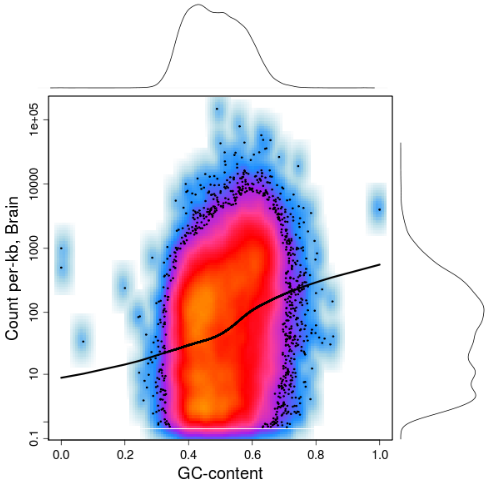


(d) Count per-kb vs. GC-content

Figure S4: *Distribution of UI gene counts for Brain, by length and GC-content.* For each gene, total and per-kb read counts were summed over the seven Brain lanes (MAQC-2). Bivariate binned Gaussian kernel density smoothers are displayed for: (a) total gene count vs. gene length; (b) per-kb gene count vs. gene length; (c) total gene count vs. gene GC-content; (d) per-kb gene count vs. gene GC-content. Marginal Gaussian kernel density smoothers are displayed above and to the right of the plots for gene length/GC-content and gene count, respectively. The curves represent lowess fits. Only genes with non-zero read counts were included. Per-kb counts have been scaled to match the range of the raw counts.

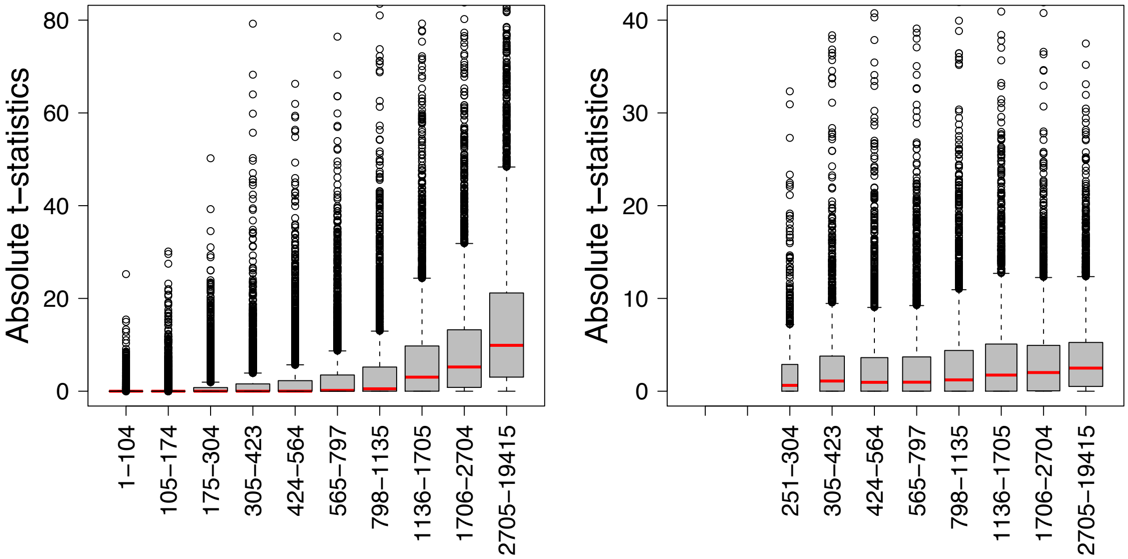


(a) Full-length UI genes

(b) 250-bp UI gene regions

Figure S5: *Differential expression statistics, by length.* Boxplots of absolute DE $t$-statistics (delta method) stratified by length for: (a) full-length genes and (b) a random sample of 250 base-pairs for each full-length gene longer than 250 base-pairs. The width of each boxplot is proportional to the number of genes within each length stratum.

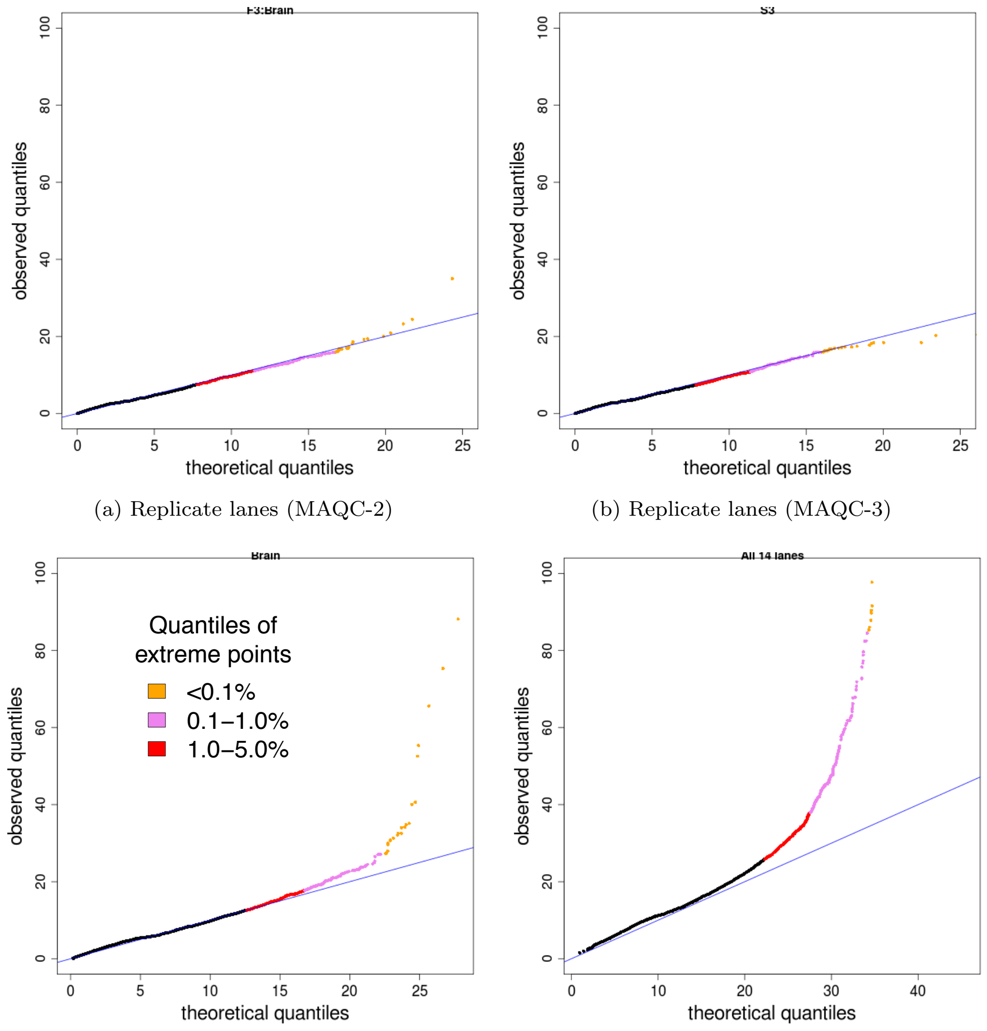


(a) Replicate lanes (MAQC-2)

(b) Replicate lanes (MAQC-3)

(c) Lanes across flow-cells (MAQC-2)

(d) Lanes across library prep and flow-cells (MAQC-3)

Figure S6: *Goodness-of-fit of gene-level multiplicative Poisson model across lanes, flow-cells, and library preparations.* The multiplicative Poisson model (GLM 1 in Table S4) is fit to the following sets of lanes representing different combinations of biological samples, library preparations, and flow-cells. Panel (a): Four replicate Brain lanes in flow-cell F3. Panel (b): Four replicate UHR lanes of library preparation S3 in flow-cell F4. Panel (c): Seven Brain lanes across flow-cells F2 and F3. Panel (d): Fourteen UHR lanes of four library preparations across flow-cells F4 and F5. Goodness-of-fit statistics are computed and displayed in $\chi^2$ quantile-quantile plots. Genes with goodness-of-fit statistics in the top quantiles of the $\chi^2$-distribution are displayed using colored plotting symbols as indicated in legend.

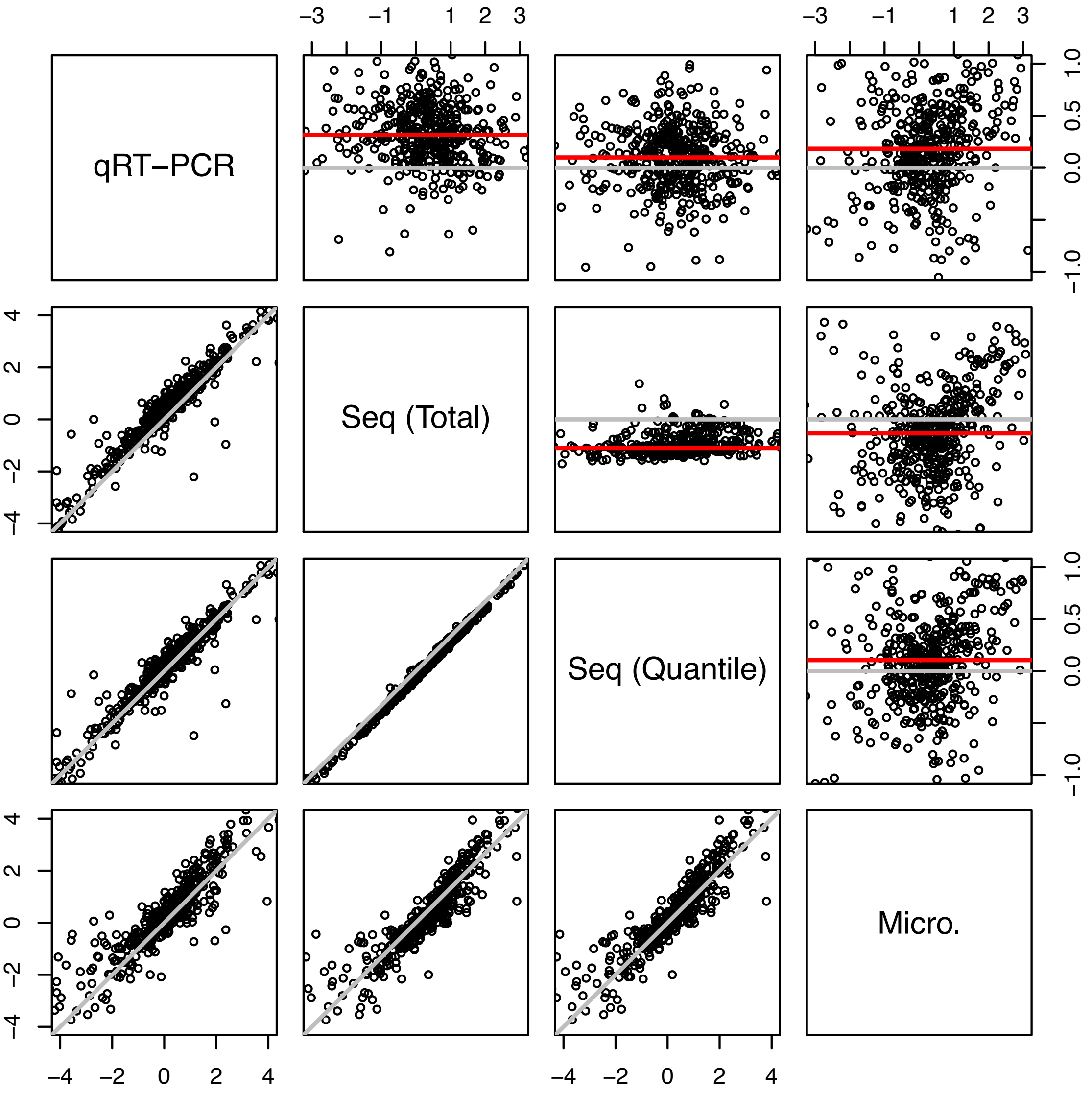


Figure S7: *Pairs plot comparing DE measures for mRNA-Seq, microarray, and qRT-PCR.* In the lower triangle are standard scatterplots of UHR to Brain log-fold-changes, while in the upper triangle are mean-difference plots of these DE measures. No difference between the DE measures is shown by a grey line; in the MD-plots, the value of the median difference is shown as a red line. Note that the difference between total-count and median normalization is just a shift of the log-ratio values and thus we show only the total-count normalization.

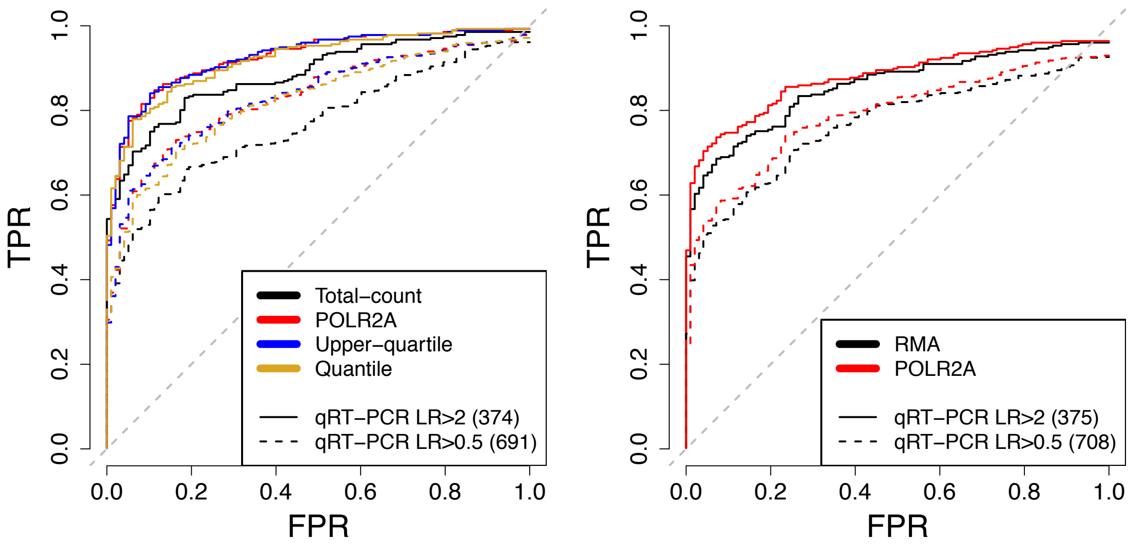


(a) mRNA-Seq

(b) Microarray

Figure S8: *Comparison of mRNA-Seq and microarray normalization procedures: ROC curves.* Panel (a): ROC curves comparing mRNA-Seq DE calls for total-count, POLR2A, upper-quartile, and quantile normalization. Panel (b): ROC curves comparing microarray DE calls for RMA and POLR2A normalization. Genes were declared DE if their qRT-PCR absolute log-ratio was greater than 2.0 (solid) or greater than 0.5 (dashed); genes were declared non-DE if their absolute log-ratio was less than 0.2.

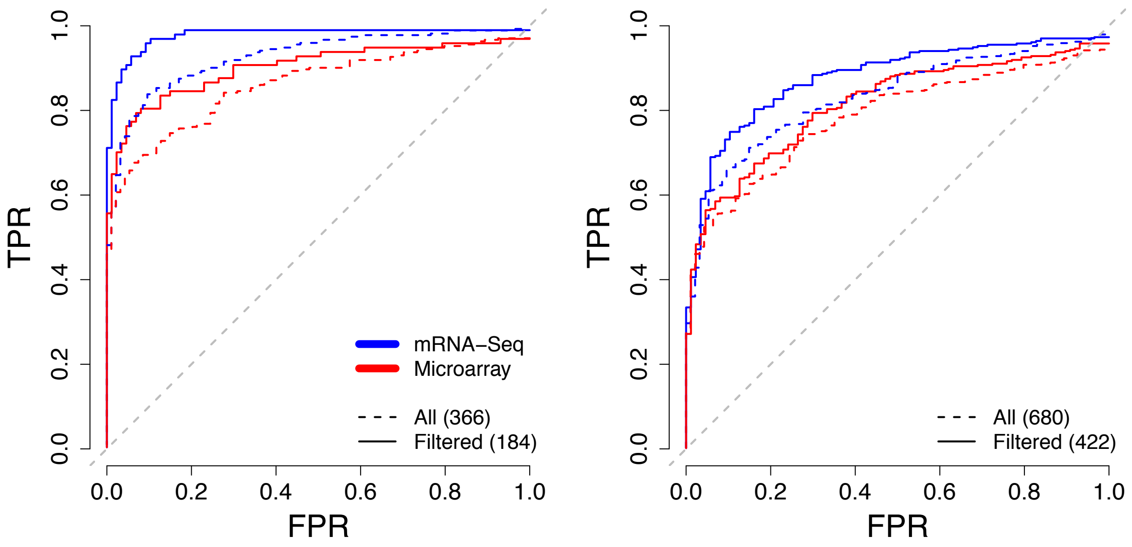


(a) LR $\geq 2$ (b) LR $\geq 0.5$

Figure S9: *Comparison of mRNA-Seq and microarray DE calls: ROC curves.* ROC curves comparing upper-quartile normalized mRNA-Seq and RMA normalized microarray DE calls. Genes were declared DE if their qRT-PCR absolute log-ratio was (a) greater than 2 or (b) greater than 0.5; genes were declared non-DE if their absolute log-ratio was less 0.2. Considered were those genes common to all three platforms. Filtering as in Figure 1.

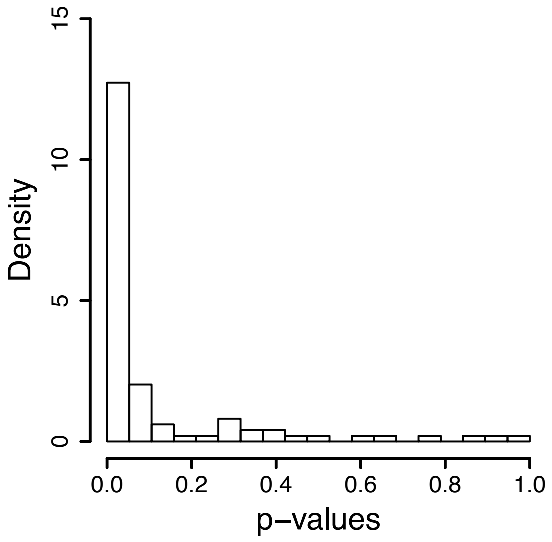


(a) Sequencing

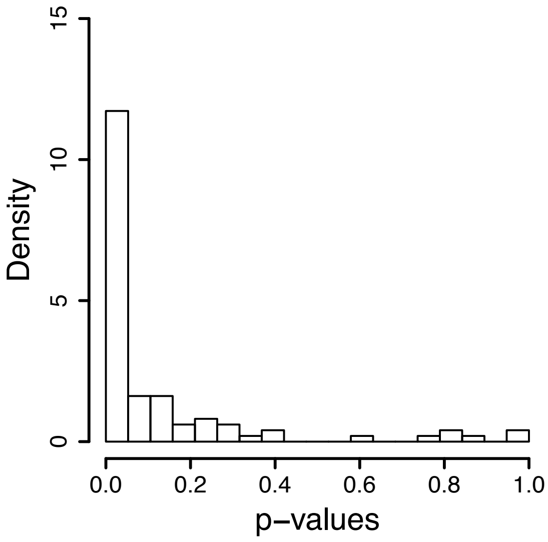


(b) Microarray

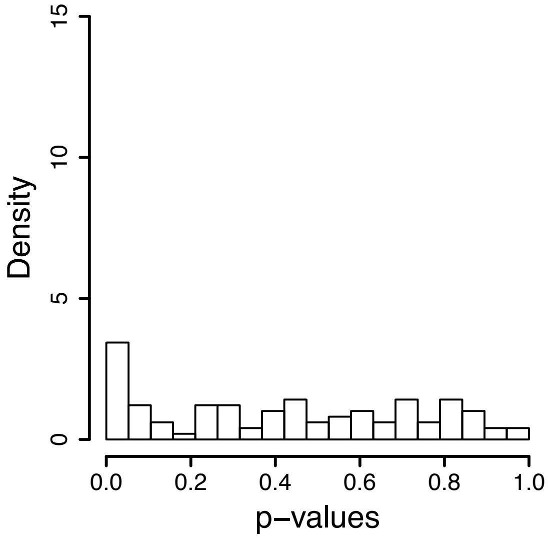


(c) Technical Replication (flowcell)

Figure S10: *Distribution of p-values for genes declared non-DE based on qRT-PCR data.* Histograms of the nominal $p$-values for testing for differential expression between UHR and Brain based on (a) mRNA-Seq data, (b) microarray data, using limma as described in Section S1.3. Panel (c) shows histogram of the nominal $p$-values for testing for flow-cell effects in the MAQC3 datase (Model 6, Table S4).
