# Supplementary material for: Evaluation of statistical methods for normalization and differential expression in mRNA-Seq experiments
Source: BMC Bioinformatics. 2010 Feb 18;11:94. doi: 10.1186/1471-2105-11-94 (PMC2838869; doi:10.1186/1471-2105-11-94)
Supplement: Additional file 2 — Supplementary Text File. Additional text to describe further details and results of the analysis. [file 1471-2105-11-94-S2.PDF]

Supplementary Text

S1 Data

S1.1 mRNA-Seq data

Sequencing reads from both MAQC-2 and MAQC-3 experiments have been deposited to the short-read archive under the accession number, SRA010153.1

The calibration method used in Bustard for quality-scoring of base-calls is highly relevant in terms of experimental design. In the auto-calibration method, base-calls are scored in a manner that is similar to the phred base-caller [17]. An alternative, recommended by Illumina, is to reserve one control lane per flow-cell for sequencing DNA, typically bacteriophage phi X genomic DNA [15].

Bustard also provides a variety of read quality measures. For a given cluster, the *chastity* c_k at cycle k is defined as the highest of the four fluorescence intensities divided by the sum of the highest two intensities. The *purity filter* (PF) discards any read for which the chastity at any of the first 12 sequencing cycles is less than 60%, i.e., $\min_{1 \leq k \leq 12} c_k < 0.60$ [15, Supplementary Information, p. 6]. For the MAQC-2 and MAQC-3 datasets, the percentage of reads passing the purity filter (out of the total number of clusters) varies between 50% and 76% per lane. Summaries of the Genome Analyzer output are provided in Tables S1 and S2.

We used Bowtie [16], Version 0.10.1, to align reads to the genome (*H. sapiens*, NCBI 37.1 assembly). We used a strict alignment policy, which enforces a strong definition of uniqueness: a perfect match is a read that perfectly matches a position and does not match elsewhere, even when allowing up to two mismatches. In this regard, we minimize the chance that a perfect match read is a read with an error that happens to perfectly match elsewhere. The Bowtie command for implementing this mapping strategy is:

```
-r -v 2 -a -m 1 -p 8 --quiet h_sapiens_37_asm
```

Mapped reads were classified into the following three nested categories: (1) *purity-filtered perfect match* (FPM) reads, that passed the purity filter and mapped uniquely as described above; (2) *purity-filtered mismatch* (FMM) reads, that passed the purity filter and mapped with either 0, 1, or 2 mismatches; (3) *mismatch* (MM) reads, that mapped with either 0, 1, or 2 mismatches, regardless of purity filtering.

As a result of the above pre-processing steps, we therefore have six sets of mapped reads, corresponding to two calibration methods (auto-calibration and phi X calibration) and three mapping stringencies (FPM, FMM, and MM). In our main analysis, we focus on phi X-calibrated, purity-filtered reads that map uniquely to the genome, with up to two mismatches (FMM).

Table S1: *MAQC-2: Pre-processing summary.* The table reports summaries from Illumina's standard Genome Analyzer pre-processing pipeline: Firecrest image analysis and Bustard base-calling [15]. "Yield (kb)": Product of number of purity-filtered clusters and number of bases per cluster (per lane). "Raw clusters": Average ± standard deviation of per-tile number of clusters detected by the image analysis module of the pipeline. "PF clusters": Average ± standard deviation of per-tile number of detected clusters that meet the purity filtering criterion. Note that the fifth lane in each flow-cell was reserved for sequencing phi X genomic DNA.

Flow-cell	Lane	Biology	Yield (kb)	Raw clusters	PF clusters
F2	L1	UHR	296866	128513 ± 8346	88353 ± 9043
F2	L2	Brain	277172	113931 ± 13407	81641 ± 13201
F2	L3	UHR	324216	134627 ± 9441	92633 ± 8700
F2	L4	Brain	294120	112663 ± 6475	84883 ± 5541
F2	L6	UHR	310230	131166 ± 8986	88637 ± 9422
F2	L7	Brain	283315	113651 ± 7401	80947 ± 8981
F2	L8	UHR	287474	122293 ± 12000	82135 ± 10298
F3	L1	Brain	203301	117128 ± 6695	58086 ± 16486
F3	L2	UHR	260693	135475 ± 7102	74483 ± 14256
F3	L3	Brain	273610	118160 ± 6825	78174 ± 10553
F3	L4	UHR	313353	136806 ± 7869	89529 ± 9365
F3	L6	Brain	288766	120813 ± 7309	82504 ± 9424
F3	L7	UHR	288312	136649 ± 7037	82374 ± 11148
F3	L8	Brain	243072	116163 ± 6596	69449 ± 10014

Note that, by mapping to the genome, we do not capture exon-exon junction reads, which would be relevant in studies of alternative splicing. In any given lane, around 10% of the reads mapped to exon-exon junctions. Additionally, the library preparation protocol does not allow consideration of strand-specific counts, i.e., reads mapping to the forward and reverse strands are pooled.

S1.2 qRT-PCR data

For benchmarking purposes, we use the *quantitative real-time polymerase chain reaction* (qRT-PCR) data of [13] to obtain distinct measures of gene expression (Gene Expression Omnibus (GEO), Series GSE5350, www.ncbi.nlm.nih.gov/geo). In this TaqMan assay, a quantitative measure of template abundance is provided by the *threshold cycle* (C_T), i.e., the number of PCR cycles at which one detects a significant exponential increase in the fluorescence of a labeled TaqMan probe. The greater the threshold cycle, the less abundant the template.

As described in [13], p. 1120–1121, for each of 997 protein-coding genes, between four (994 genes) and eight (3 genes) C_T measures were obtained for each of Brain and UHR. Due to annotation differences, of the 997 genes assayed by qRT-PCR, 965 matched a unique UI gene. We find there is no systematic

Table S2: *MAQC-3: Pre-processing summary.* Cf. Table S1 caption.

Flow-cell	Lane	Lib. Prep.	Yield (kb)	Raw clusters	PF clusters
F4	L1	S3	258032	114634 ± 10132	79272 ± 13356
F4	L2	S4	334535	144311 ± 11288	96547 ± 10952
F4	L3	S3	311489	120708 ± 9614	88997 ± 9017
F4	L4	S4	354932	141855 ± 11771	101409 ± 8263
F4	L6	S3	316489	119383 ± 8989	90425 ± 7053
F4	L7	S4	336469	140959 ± 10373	97105 ± 7050
F4	L8	S3	278196	113575 ± 8529	79484 ± 8161
F5	L1	S5	251885	118150 ± 7715	81780 ± 8955
F5	L2	S6	364813	162401 ± 11096	105285 ± 4996
F5	L3	S5	324904	124526 ± 7446	92829 ± 3300
F5	L4	S6	371195	158799 ± 11354	106055 ± 3718
F5	L6	S5	314057	118976 ± 6562	89730 ± 2854
F5	L7	S6	357717	157457 ± 9475	102204 ± 4237
F5	L8	S5	288629	122585 ± 10022	82465 ± 8166

relationship between gene expression measures and mapping status.

Following [13], a detection limit of 35 was set on the raw C_T values. For each type of biological sample (Brain and UHR), genes were further classified as present (P) if they were detectable in at least three fourths of the qRT-PCR assays and absent (A) otherwise. According to this criterion, 797 genes were declared present in both Brain and UHR samples, 26 present in only Brain samples, 76 present in only UHR samples, and 40 absent in both types of samples. The C_T measures available from GEO were normalized as in [13], separately for the Brain and UHR samples, using the POLR2A gene as a reference. In what follows, the qRT-PCR expression measures are represented as

$$Y_{i,j} \equiv \Delta C_{i,j} \times \log 2, \quad \text{(S-1)}$$

where $\Delta C_{i,j} = C_{i,POLR2A} - C_{i,j}$ are POLR2A-normalized threshold cycles C_T for protein-coding genes $j = 1, \ldots, 939$, in TaqMan assays $i = 1, \ldots, n_j$ ($n_j = 8$ for all but three genes that have $n_j = 16$). The qRT-PCR measures are originally on a log base-2 scale. Multiplication by $\log 2$ transforms these measures to the natural logarithmic scale used throughout. The qRT-PCR estimate of UHR to Brain expression log-fold-change is the difference of averages: $\bar{Y}_{UHR,j} - \bar{Y}_{Brain,j}$.

S1.3 Affymetrix microarray data

Affymetrix microarray data were downloaded from GEO (GSE5350, MAQC_AFX_123456_120CELs.zip). To minimize variation across labs, we used data only from lab 1, i.e., AFX_1_[A—B][1-5].CEL$. Arrays

were pre-processed using RMA [23] and then differential expression was determined by the R/Bioconductor package limma [24], using the standard pipeline of `lmFit` and `eBayes`.

In order to match Affymetrix probesets with our UI genes, we used the R/Bioconductor package biomaRt, which retrieves data from Ensembl, Version 55. In cases where multiple probesets matched to a single UI gene, we took the median measurement for the log-ratio, standard errors, and p-values, so as not exclude a large fraction of the microarray data.

S2 Defining genomic regions of interest

Using Ensembl, Version 55, annotation, we define a *union-intersection (UI) gene* as a composite gene consisting of unions of constitutive exons (including UTRs) that do not overlap a coding or UTR region of another gene. Specifically, for a given gene, a *constitutive exon* is defined as a set of consecutive exonic bases (i.e., portion of or entire exon) that belong to each isoform of the gene of interest. We further exclude any portion of such region that overlaps the coding or UTR region of any other gene, either constitutive or alternative, on either strand (Figure SS1a). A gene model defined according to this union-intersection principle can be viewed as representing all isoforms of a given gene. Reads are assigned to a given gene if their 5'-end falls within the region, as depicted in Figure SS1b.

Figure SS2 examines basic features of the set of UI genes considered in the present article and built using gene annotation from Ensembl (www.ensembl.org, Version 55). Figure SS4 displays an example gene and its base-level read counts. In Figure SS3, observed counts for the UI genes are reported.

We also define an Ensembl gene as the union of all exons from a given gene, excluding regions which overlap any other gene on either strand. Our definition of UI genes is clearly more restrictive than that of Ensembl genes, as it retains only constitutive exons. The genome coverage of UI genes is 42,708,318 base-pairs, whereas the coverage of Ensembl genes is 82,020,267 base-pairs.

We call an Ensembl gene or a UI gene present, if it has at least one read in both Brain and UHR samples. Filtered genes are defined as having at least 20 reads in both samples.

S3 Generalized linear models for gene-level counts

Consider J genes and let $X_{i,j}$ denote the number of reads mapping to gene j in lane i. Sums of counts over all lanes or genes are represented with the standard "$\cdot$" symbol, e.g., $X_{i,\cdot}$ denotes total counts in lane i. Generalized linear models (GLM) provide a flexible and extensible statistical inference framework for mRNA-Seq. Though we focus on models with the *log/Poisson link* function [25], what follows may be

applied using alternative link functions and distributions, such as the negative binomial.

We formulate a gene-level GLM for read counts $X_{i,j}$, such that $\log(\mathrm{E}[X_{i,j}]) = \lambda_{a(i),j} + \theta_{i,j}$, where $a(i) \in \{1, \ldots, A\}$ is the biological group (e.g., Brain or UHR) corresponding to lane i, $\lambda_{a(i),j}$ is the parameter of interest representing the expression level of gene j in biological group $a(i)$, and $\theta_{i,j}$ is a nuisance parameter representing experimental effects, such as library preparation, flow-cell, and lane effects. Suitable identifiability constraints need to be specified for each experimental design under consideration, e.g., $\sum_j \exp(\lambda_{a,j}) = 1$ for each biological group a.

Ultimately, the parameter of interest is the ratio of transcript counts in biological group a_2 vs. a_1, i.e., a transcript expression fold-change. In the mRNA-Seq assay, each transcript is divided into a number of fragments. As a result, the parameter $\exp(\lambda_{a_2,j} - \lambda_{a_1,j})$ represents the ratio of the number of fragments for biological group a_2 vs. a_1. Under certain assumptions for the library preparation process (concerning fragmentation, in particular), it can be argued that transcript and fragment fold-changes are proportional, with a single proportionality constant across all genes.

It is clear any reasonable model must normalize read counts to adjust for the large differences in sequencing depths between lanes (or samples). This can be achieved by introducing a lane-level parameter, δ_i, in the GLM,

$$\log(\mathrm{E}[X_{i,j}]) = \delta_i + \lambda_{a(i),j} + \theta_{i,j}. \tag{S-2}$$

Instead of fitting the above GLM jointly to all J genes (in the tens of thousands), it is equivalent to fit the following log-linear regression model per gene,

$$\log(\mathrm{E}[X_{i,j}|d_i]) = \log d_i + \lambda_{a(i),j} + \theta_{i,j}, \tag{S-3}$$

where d_i is a lane-level random variable, such as the total lane count $X_{i,\cdot}$, and the offset $\log d_i$ is to be treated as a quantitative covariate whose regression coefficient is set to one.

To evaluate the presence of experimental effects, we fit the model of Equation (1) with different choices of $\theta_{i,j}$ that account for groupings of lanes into flow-cells (fc) or library preparations (prep), as well as interactions of these effects with biological (bio) effects, where appropriate (see Table S4). We use likelihood ratio statistics per gene to compare the fits of models with various combinations of effects, e.g., (1+bio+fc) vs. (1+bio). We also use χ^2 goodness-of-fit statistics to assess deviation from a particular null model – again per gene (such an approach was also applied in [6], to assess goodness-of-fit for a particular model of inter-lane variation).

We note that by estimating a large number of parameters we risk overfitting and introducing noise into our estimators. In this instance, however, our goal is an overall assessment of experimental effects. To correct for such effects in practice and obtain reliable estimators of $\lambda_{a,j}$ for specific genes, more sophisticated approaches may be appropriate, such as pooling data across genes using empirical Bayes methods.

S4 Normalization

The total-count, upper-quartile, and POLR2A normalization procedures involve a choice of a global scaling factor d, which is a vector of length equal to the number of lanes (14 in each of the two MAQC datasets). For total-count normalization, the value d_i for lane i was the total number of mapped reads for lane i, where mapped here means mapped to a genomic location. Upper-quartile normalization was based on the constitutive gene counts, described above, but excluding any gene that had zero counts for all of the lanes. Then for each lane, d_i was the upper-quartile (75 percentile) of all the gene counts in lane i. For POLR2A normalization, d_i is the number of reads mapped to the constitutive regions of POLR2A for lane i. The offset d is incorporated in GLM-based tests (i.e., the LLR and the t-statistics) as described below. We note that the total-count offset corresponds to the maximum likelihood estimator of the parameter δ_i in the GLM of Equation (S-2).

We rescale the offset vector d so that the sum of its elements is equal to the total count across all lanes (roughly 67 million). This is done solely for the purpose of comparing normalizing factors and only affects Fisher's exact test, for which the distribution of the test statistic depends on the actual magnitude of d. The GLM-based tests are unaffected, because differences in the overall magnitude of the normalizing scale factor can be absorbed into an intercept term.[1] In our implementation of Fisher's exact test, d is acting as observed data even though it is not (except in the case of total-count normalization). GLM clearly give a more logical framework for allowing different choices of global normalization.

The final normalization considered is quantile normalization [18], as implemented in the R package aroma.light [26]; the median across sorted lanes was chosen as the reference distribution. The normalized data are rounded to produce integer values that can be used with each of the DE statistics described below.

S5 Differential expression statistics

Identifying genes that are differentially expressed between A conditions corresponds to testing the following J per-gene null hypotheses: $H_0(j) : \lambda_{1,j} = \cdots = \lambda_{A,j}$, where $\lambda_{a,j}$ is the expression level of gene j in

[1]The GLM of Equations (S-2) and (S-3) do not include an intercept term, but this is just a matter of reparameterization.

samples of type a. We evaluate three main types of DE tests.

- **Log-likelihood ratio (LLR) statistics for GLM**:

$$T_j^{LLR} = 2(\mathrm{l}_j(\hat{\lambda}, \hat{\theta}) - \mathrm{l}_j(\hat{\lambda}^0, \hat{\theta}^0)) \dot{\sim} \chi^2(A-1), \tag{S-4}$$

  where l_j denotes the log-likelihood function for the jth gene and $(\hat{\lambda}, \hat{\theta})$ and $(\hat{\lambda}^0, \hat{\theta}^0)$ denote, respectively, the maximum likelihood estimators (MLE) of the biological and experimental effect parameters under the full model and null model.

- **t-statistics for GLM (2 sample comparisons, $A = 2$)**:

$$T_j^t = \frac{(\hat{\lambda}_{2,j} - \hat{\lambda}_{1,j}) - 0}{\sqrt{\widehat{\mathrm{Var}}[\hat{\lambda}_{1,j}] + \widehat{\mathrm{Var}}[\hat{\lambda}_{2,j}]}} \dot{\sim} N(0, 1), \tag{S-5}$$

  where the variances of $\hat{\lambda}$ may be estimated from (1) the standard GLM fitting procedure `glm` in R [27], e.g., based on an estimator of the information matrix obtained from the Hessian of the log-likelihood function, or (2) the delta method, where $\widehat{\mathrm{Var}}[\hat{\lambda}_{a,j}] = 1/\sum_i \mathrm{I}(a(i) = a) X_{i,j}$ (assuming $\theta_{i,j}$ constant across samples).

- **Fisher's exact test** is based on the $2 \times A$ contingency table created by cross-tabulating genes with biological sample type (Brain and UHR). The Mantel-Haenszel test of conditional independence within stratum extends Fisher's exact test to account for a single additional experimental effect (e.g., flow-cell). In all cases, except quantile normalization, row 1 corresponds to the total number of reads observed in the jth gene; in quantile normalization, it corresponds to the rounded quantile-normalized value. For total-count normalization, row 2 corresponds to the lane totals less the number of reads in the jth gene. In the case of POLR2A, upper-quartile, and quantile normalization, pseudo-counts are generated to match the total number of reads (see Section S4). The tests are implemented using the `fisher.test` and `mantelhaen.test` functions in R.

S6 Receiver operator characteristic curves

Receiver operator characteristic curves (ROCs) were based on the set of common genes for the sequencing and qRT-PCR data. Additionally, only genes that were called present on both the qRT-PCR platform and the sequencing platform were considered. For the sequencing data, this required that the genes have at least one read in any of the 14 lanes of sequencing. For the definition of present/absence on the qRT-PCT

platform, see Section S1 above. In the ROCs presenting microarray results, we further required the gene to be present on the microarray platform.

Definition of DE based on qRT-PCR data Conceivably, every gene could be declared differentially expressed at some cutoff, which means any "false positive" could be due either to noise, errors, or extremely high sensitivity of the (sequencing or microarray) platform. Furthermore, the qRT-PCR measures of DE are themselves imperfect, though generally accepted as the best available – they have very low levels of variation and the variation is extremely uniform across genes. Rather than rely on the p-values from a test statistic for differential expression in qRT-PCR, we instead remove the 12 genes with standard errors greater than .25. In this manner, we focus on the more biologically relevant fold-change rather than the standard errors. We divide the genes assayed by qRT-PCR into three sets, "non-DE", "DE", and "no-call", based on whether their absolute expression log-fold-change is less than a, greater than b, or falls within the interval $[a, b]$, respectively. We ignore the "no-call" genes when determining true/false positives/negatives. In all of the plots presented here, $a = 0.20$, though lower values were also explored with no change in the results.

To ensure that focusing on fold-change, as opposed to p-value, does not change our overall conclusions, we examine the performance of both upper-quartile normalization and the different test statistics using the qRT-PCR p-values to declare DE. A gene is declared DE based on the qRT-PCR if its Bonferoni adjusted p-value is below 0.05 (out of a total of 722 genes common among three platforms). Table S3 compares the DE calls, as determined using the Bonferoni adjusted p-value and log-fold-change cutoffs (see also Figure SS9 for density plots). The non-DE calls made based on log-fold-change were also non-DE based on p-value for all but 5 genes. Similarly, for DE calls, most of the genes called DE for the log-fold-change cutoff are also DE for the p-value cutoff, especially for large values of log-fold-change. Finally, to validate the general conclusions, we constructed ROC plots which demonstrate that, although shifted, the comparative performance of the methods is the same as that determined by qRT-PCR log-fold-change. In Figure SS5, upper-quartile normalization performs better than total-count normalization and likelihood ratio tests as well as Fisher's exact tests perform better than their t-statistic counterparts, recapitulating what we see in the ROC curves based on log-fold-change cutoffs.

Definition of true and false positive rates Given a "DE" (positive, P) or "non-DE" (negative, N) call from qRT-PCR, define a true positive (TP) as the event that the test of interest (based on either sequencing or microarray data) calls a gene DE that qRT-PCR called DE and that the direction of DE agrees between

Table S3: *qRT-PCR gene agreement between cutoffs based on p-value and fold-change.* Genes determined DE by absolute log-fold-change as compared to genes declared DE by Bonferroni adjusted nominal p-values.

			Bonferroni p-value		
			non-DE $> .05$	DE $\leq .05$	Total # of genes
	non-DE	< 0.2	89	5	94
absolute log-fold-change	no-call	$[0.2, 0.5]$	50	42	92
		$(0.5, 0.75]$	26	79	105
	DE	$(0.75, 2]$	27	182	209
		> 2	18	254	272
	Total # of genes		210	562	772

the two assays. Let a false positive (FP) event occur when the test calls a gene DE that qRT-PCR called non-DE (Table 1). We consider a true positive rate (TPR) defined as

$$\Pr(\text{TP}|\text{qRT-PCR is DE}) = \frac{\Pr(\text{TP}, \text{qRT-PCR is DE})}{\Pr(\text{qRT-PCR is DE})}$$

and estimated with

$$\frac{(\#\text{ TP and qRT-PCR is DE})/(\text{total }\#\text{ genes})}{(\#\text{ qRT-PCR is DE})/(\text{total }\#\text{ genes})} = \frac{\text{TP}}{\text{P}}.$$

Note that this is not the standard definition of TPR, usually expressed in terms of TP, FP, TN, and FN. We consider the standard definition of false positive rate (FPR),

$$\Pr(\text{FP}|\text{qRT-PCR is non-DE}),$$

estimated with

$$\frac{(\#\text{ FP and qRT-PCR is non-DE})/(\text{total }\#\text{ genes})}{(\#\text{ qRT-PCR is non-DE})/(\text{total }\#\text{ genes})} = \frac{\text{FP}}{\text{N}}.$$

S7 Experimental effects: Lane, flow-cell, and library preparation

We investigate various experimental effects for gene-level counts, including lane, flow-cell, and library preparation effects. For this, we rely on the total-count normalization, which gives the best results in terms of goodness-of-fit of the Poisson model for replicate lanes. Figure SS6 displays mean-difference scatterplots of expression fold-changes vs. overall expression measures for lanes representing different combinations of flow-cells, library preparations, and biological groups (Brain and UHR). It is immediately clear that the magnitude of the differences between biological groups dwarfs any of the experimental effects.

Mean-difference scatterplots of log-fold-change vs. overall expression are preferable to scatterplots of expression measures, as the latter often give a misleading impression of concordance between samples.

Replicate lanes Figures S6a and S6b show quantile-quantile (QQ) plots of χ^2 goodness-of-fit statistics for the multiplicative Poisson model fit within sets of replicate lanes for each UI gene (GLM 1, Table S4). Note that zero-read genes have undefined χ^2-statistics and are not plotted. Each QQ-plot is very close to the 0, 1 line; in particular, at worst only the top 0.1% of genes (and less than 10 genes for many of the sets of replicates) do not closely follow the null distribution – a remarkably good fit for non-simulated data. When goodness-of-fit is assessed without correcting for differences in total number of reads, the results unsurprisingly show lack-of-fit. Analogous QQ-plots stratified by read count for MAQC-3 (Figure SS7) indicate that genes with a reasonable number of reads (average of 3 or more reads per lane) show excellent fit; genes with fewer reads exhibit poor fit. This discrepancy most likely results from the breakdown of the asymptotic χ^2 approximation.

Flow-cell and library preparation effects We assess whether different aspects of the experimental design (flow-cell, library preparation) influence our ability to estimate the biological effects of interest. In Figures S6c and S6d, we see that when we ignore flow-cell or library preparation designation, the QQ-plots demonstrate lack-of-fit as compared to similar plots for replicate lanes. In particular, flow-cell and library preparation QQ-plots show deviation for the top 1% and 5% of genes, respectively, whereas analogous plots for replicate lanes only show deviation in the top 0.1% (if at all). Explicitly adjusting for flow-cell or library preparation effects results in near linear QQ-plots.

Next, to assess the significance of technical effects compared to biological effects, we compare various parameterizations of the log-linear regression model using likelihood ratio statistics (Table S4). The count-stratified QQ-plots of Figure SS8a demonstrate that globally, the most significant differences between models are related to biology, as opposed to flow-cell.

Figure 4 demonstrates that flow-cell effects are much smaller in magnitude than biological effects. Although a direct comparison of library preparation effects to flow-cell and biological effects is not possible (due to confounding and nesting in MAQC-2 and MAQC-3, respectively), the boxplots suggest that both technical effects are much smaller than biological effects.

In summary, the above analysis suggests that there are both flow-cell and library preparation effects, but of less significance and of smaller magnitude than biological effects. Ignoring flow-cell has only a minor impact in detecting extremely small biological differences; almost none when genes have greater than 3 reads/lane.

Table S4: *Log-linear regression models.* The following class of log-linear regression models are considered separately for each gene j: $\log(\mathrm{E}[X_{i,j}|X_{i,\cdot}]) = \log X_{i,\cdot} + \lambda_{a(i),j} + \theta_{i,j}$. Each row in the table corresponds to a different parameterization of the biological effect λ (bio) and experimental effect θ, to represent different combinations of biological, library preparation, and flow-cell effects. Specifically, library preparation (prep) and flow-cell (fc) effects are denoted, respectively, by $\beta_{b(i)}$ and $\gamma_{c(i)}$, where $a(i)$, $b(i)$, and $c(i)$ map lane i to its corresponding biological, library preparation, or flow-cell group, respectively. Recall that in MAQC-2, biological effects (λ) are confounded with library preparation effects (β), and in MAQC-3, library preparation effects (β) are nested within flow-cell effects (γ). The gene index j is omitted to simplify notation.

Dataset	Model	Formula	$\lambda_{a(i)}$	θ_i	# parameters	Constraints
MAQC-2	1	1	0	α	1	$\lambda_{Brain} = 0$, $\gamma_{F2} = 0$
	2	1 + bio	$\lambda_{a(i)}$	α	2	
	3	1 + fc	0	$\alpha + \gamma_{c(i)}$	2	
	4	1 + bio + fc	$\lambda_{a(i)}$	$\alpha + \gamma_{c(i)}$	3	
MAQC-3	5	1	0	α	1	$\beta_{S3} = 0$, $\gamma_{F4} = 0$
	6	1 + fc	0	$\alpha + \gamma_{c(i)}$	2	
	7	1 + fc:prep	0	$\alpha + \beta_{b(i)}$	4	

S8 Phi X calibration analysis

In each flow-cell, one lane out of eight was reserved for sequencing bacteriophage phi X genomic DNA and used by Genome Analyzer's base-caller Bustard for base-calling and quality-scoring [15, Supplementary Information, p. 7]. This practice has important experimental design implications, in terms of sample size and balance. We used the MAQC-2 dataset to investigate the impact of the calibration method (phi X calibration vs. auto-calibration) at various levels of the analysis pipeline, including base-calling, read-mapping, and (differential) expression inference.

S8.1 Base-calling and quality-scoring

We first examine the effect of the calibration method on base-calls by cycle and by lane (in base-calling, a cycle refers to a position in a read, here, from 1 to 35).

The pseudo-color image in Figure SS10 illustrates that there is good overall agreement between phi X and non-phi X-calibrated reads (less than 3% discrepancy). However, the discrepancy rate between the base-calls for the two calibration methods varies between cycles (higher for later cycles) and between lanes and flow-cells (higher for flow-cell F3). Furthermore, Figure SS11 shows that not all base substitutions are equally likely, with phi X calls of 'C' being more frequently assigned another base by auto-calibration and the 'C' to 'G' transversion being the most common substitution.

Overall, quality scores assessing the base-calls tend to be higher with auto-calibration. Figure SS12a shows

per-cycle quality scores for phi X and non-phi X-calibrated reads averaged across the seven lanes of each flow-cell. The quality scores for auto-calibration are generally higher at each cycle and, as previously noted, quality degrades through cycle [15]. Flow-cell F3 generally has lower quality scores and much steeper drops in quality for higher cycles. Additionally, Figure SS12b, which shows the difference in quality scores by lane, demonstrates substantial variation in differences of quality scores between flow-cells and between lanes within flow-cells. The differences in base-calling quality scores between flow-cells F2 and F3 may explain the flow-cell effects reported earlier on downstream gene expression measures.

S8.2 Absolute and relative expression measures

Next, we consider the impact of the calibration method on (differential) expression statistics, based on purity-filtered perfect match (FPM) reads.

The significance of differences in estimates between the two calibration methods can be assessed by comparing observed differences to a permutation distribution of differences obtained by randomly swapping the auto-calibrated and phi X-calibrated sets of read counts for each of the 14 lanes. Such a permutation scheme respects the joint distribution of gene counts within lane and the experimental design (lane/flow-cell/library preparation/biological sample structure). The empirical cumulative distribution function (ECDF) and scatterplots of permutation p-values in Figure SS13 suggest that, although small in magnitude, the differences in absolute expression measures are significant, especially for ROI with large read counts (Figure SS13, Panels (a) and (c)). However, differences in expression fold-changes between UHR and Brain do not appear to be significant (Figure SS13, Panels (b) and (d)). Finally, in Figure SS14, we note that, as expected, DE statistics determined using auto-calibration tend to be more extreme due to the larger number of counts with auto-calibration. However, this difference is insubstantial for a majority of genes.

In summary, while there are some differences between phi X and auto-calibration in the early stages of the analysis pipeline, the differences in terms of differential expression are small. Unfortunately, we only have two flow-cells from which to assess the impact of auto-calibration vs. phi X calibration. However, it seems quite clear, using these two flow-cells, that auto-calibration is advantageous, as it yields more balanced designs, frees up one lane per flow-cell, and produces a larger number of higher quality reads per lane.

References

1. Chiang DY, Getz G, Jaffe DB, O'Kelly MJT, Zhao X, Carter SL, Russ C, Nusbaum C, Meyerson M, Lander ES: **High-resolution mapping of copy-number alterations with massively parallel sequencing**. *Nature Methods* 2009, **6**:99–103.

2. Dohm JC, Lottaz C, Borodina T, Himmelbauer H: **Substantial biases in ultra-short read data sets from high-throughput DNA sequencing**. *Nucleic Acids Research* 2008, **36**(16):e105.

3. Hoen PAC, Ariyurek Y, Thygesen HH, Vreugdenhil E, Vossen RHAM, de Menezes RX, Boer JM, van Ommen GJB, den Dunnen JT: **Deep sequencing-based expression analysis shows major advances in robustness, resolution and inter-lab portability over five microarray platforms**. *Nucleic Acids Research* 2008, **36**(21):e141.

4. Lee A, Hansen KD, Bullard J, Dudoit S, Sherlock G: **Novel low abundance and transient RNAs in yeast revealed by tiling microarrays and ultra high–throughput sequencing are not conserved across closely related yeast species**. *PLoS Genetics* 2008, **4**(12):e1000299.

5. Li H, Lovci MT, Kwon YS, Rosenfeld MG, Fu XD, Yeo GW: **Determination of tag density required for digital transcriptome analysis: Application to an androgen-sensitive prostate cancer model**. *PNAS* 2008, **105**(51):20179–20184.

6. Marioni JC, Mason CE, Mane SM, Stephens M, Gilad Y: **RNA-seq: An assessment of technical reproducibility and comparison with gene expression arrays**. *Genome Research* 2008, **18**(9):1509–1517.

7. Mortazavi A, Williams BA, McCue K, Schaeffer L, Wold B: **Mapping and quantifying mammalian transcriptomes by RNA-Seq**. *Nature Methods* 2008, **5**(7):621–628.

8. Nagalakshmi U, Wang Z, Waern K, Shou C, Raha D, Gerstein M, Snyder M: **The transcriptional landscape of the yeast genome defined by RNA sequencing**. *Science* 2008, **320**(5881):1344–1349.

9. Wang ET, Sandberg R, Luo S, Khrebtukova I, Zhang L, Mayr C, Kingsmore SF, Schroth GP, Burge CB: **Alternative isoform regulation in human tissue transcriptomes**. *Nature* 2008, **456**(7221):470–476.

10. MAQC Consortium: **The MicroArray Quality Control (MAQC) project shows inter-andintraplatform reproducibility of gene expression measurements**. *Nature Biotechnology* 2006, **24**(9):1151–1161.

11. Oshlack A, Wakefield MJ: **Transcript length bias in RNA-seq data confounds systems biology**. *Biology Direct* 2009, **4**(14).

12. Illumina: *Sequencing Analysis Software User Guide For Pipeline Version 1.3 and CASAVA Version 1.0 T*. Illumina, Inc. 2008, [icom.illumina.com/icom/software.ilmn?id=277]. [Part # 1005359 Rev. A].

13. Canales RD, Luo Y, Willey JC, Austermiller B, Barbacioru CC, Boysen C, Hunkapiller K, Jensen RV, Knight CR, Lee KY, Ma Y, Maqsodi B, Papallo A, Peters EH, Poulter K, Ruppel PL, Samaha RR, Shi L, Yang W, Zhang L, Goodsaid FM: **Evaluation of DNA microarray results with quantitative gene expression platforms**. *Nature Biotechnology* 2006, **24**(9):1115–1122.

14. Illumina: *Preparing Samples for Sequencing mRNA*. Ilumina, Inc. 2009, [icom.illumina.com/icom/software.ilmn?id=277]. [Part # 1004898 Rev. A].

15. Bentley DR, Balasubramanian S, Swerdlow HP, et al.: **Accurate whole human genome sequencing using reversible terminator chemistry**. *Nature* 2008, **456**(7218):53–59.

16. Langmead B, Trapnell C, Pop M, Salzberg SL: **Ultrafast and memory-efficient alignment of short DNA sequences to the human genome**. *Genome Biology* 2009, **10**(3):R25.

17. Ewing B, Green P: **Base-calling of automated sequencer traces using phred. II. Error probabilities**. *Genome Research* 1998, **8**(3):186–194.

18. Irizarry RA, Hobbs B, Collin F, Beazer-Barclay YD, Antonellis KJ, Scherf U, Speed TP: **Exploration, Normalization, and Summaries of High Density Oligonucleotide Array Probe Level Data**. *Biostatistics* 2003, **4**(2):249–264.

19. Taub MA: **Analysis of high-throughput biological data: some statistical problems in RNA-seq and mouse genotyping**. *PhD thesis*, Department of Statistics, UC Berkeley 2009.

20. Durinck S, Bullard J, Spellman PT, Dudoit S: **GenomeGraphs: integrated genomic data visualization with R**. *BMC Bioinformatics* 2009, **10**:Article 2.

21. Lu J, Tomfohr JK, Kepler TB: **Identifying differential expression in multiple SAGE libraries: an overdispersed log-linear model approach.** *BMC Bioinformatics* 2005, **6**:165.

22. Robinson MD, Smyth GK: **Moderated statistical tests for assessing differences in tag abundance.** *Bioinformatics* 2007, **23**(21):2881–2887.

23. Irizarry RA, Hobbs B, Collin F, Beazer-Barclay YD, Antonellis KJ, Scherf U, Speed TP: **Exploration, normalization, and summaries of high density oligonucleotide array probe level data.** *Biostatistics* 2003, **4**(1465-4644 (Print)):249–64.

24. Smyth GK: **Linear Models and Empirical Bayes Methods for Assessing Differential Expression in Microarray Experiments**. *Statistical Applications in Genetics and Molecular Biology* 2004, **3**.

25. McCullagh P, Nelder JA: *Generalized Linear Models*. No. 37 in Monographs on Statistics and Applied Probability, Chapman & Hall/CRC, 2nd edition 1989.

26. Bengtsson H: *aroma.light: Light-weight methods for normalization and visualization of microarray data using only basic R data types* 2009, [http://www.braju.com/R/]. [R package version 1.11.2].

27. R Development Core Team: *R: A Language and Environment for Statistical Computing*. R Foundation for Statistical Computing, Vienna, Austria 2008, [www.R-project.org]. [ISBN 3-900051-07-0].

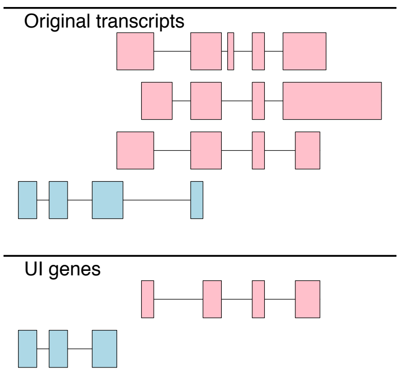

(a) UI vs. Ensembl genes

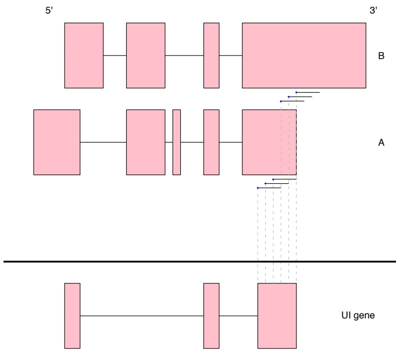

(b) UI genes and read-counting

Figure SS1: *Union-intersection and Ensembl gene models.* Panel (a): Illustration of union-intersection (UI) and Ensembl gene definitions for two genes (pink and blue) with multiple isoforms (see Section S2). The original transcripts, as would be reported by Ensembl, are displayed in the top panel. Below are the corresponding UI and Ensembl gene models. Note that because the genes overlap, the entire exon region is removed, not just the overlap. Panel (b): Illustration of read-counting for a gene with two isoforms. Isoform A has a shorter 3'-most exon as compared to Isoform B. The UI gene model includes the entire 3'-most exon for Isoform A. In addition to reads originating from the constitutive portion of the UI gene, reads emanating exclusively from Isoform B may also be counted.

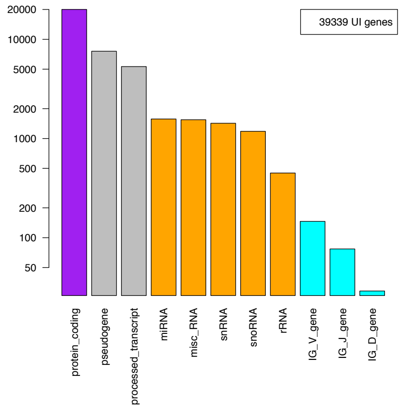

(a) Ensembl annotation

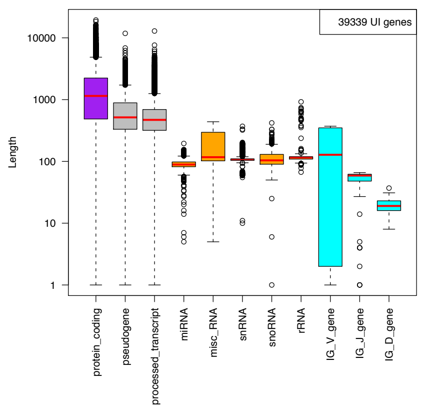

(b) Length by Ensembl annotation

Figure SS2: *UI gene Ensembl annotation.* Panel (a): Barplots of the distribution of UI genes by Ensembl annotation. Panel (b): Boxplots of UI gene lengths by Ensembl annotation. Ensembl annotation categories are sorted in decreasing order of their cardinalities; only categories comprising more than ten UI genes are displayed (11 out of 25 categories).

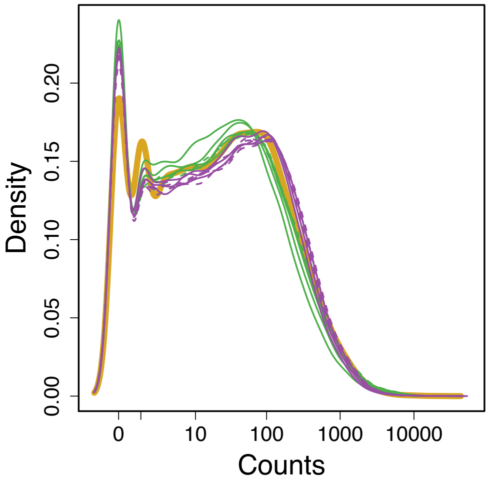

(a) Raw counts

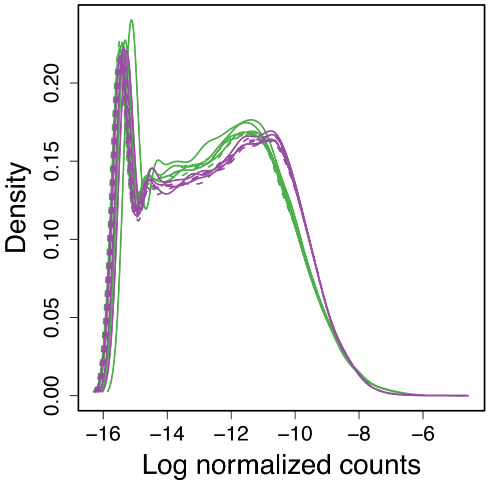

(b) Total-count normalization

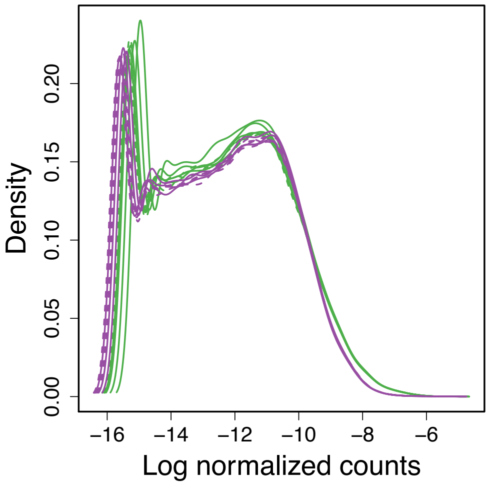

(c) Upper-quartile normalization

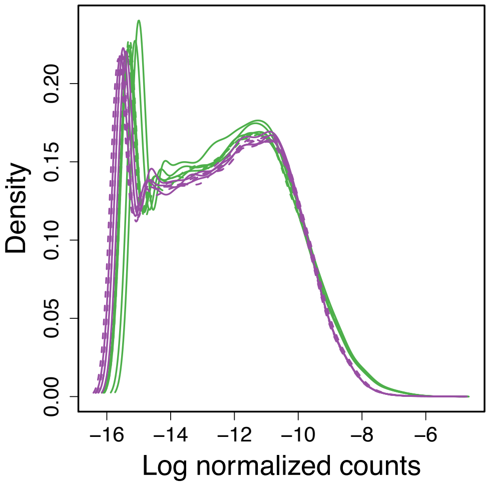

(d) POLR2A normalization

Figure SS3: *Distribution of UI gene counts.* (a) Raw counts, (b) log counts after total-count normalization, (c) log counts after upper-quartile normalization, (d) log counts after POLR2A normalization. The density of quantile-normalized counts is the same for each lane and shown in yellow in panel (a). Brain samples are shown in green and UHR samples are shown in purple.

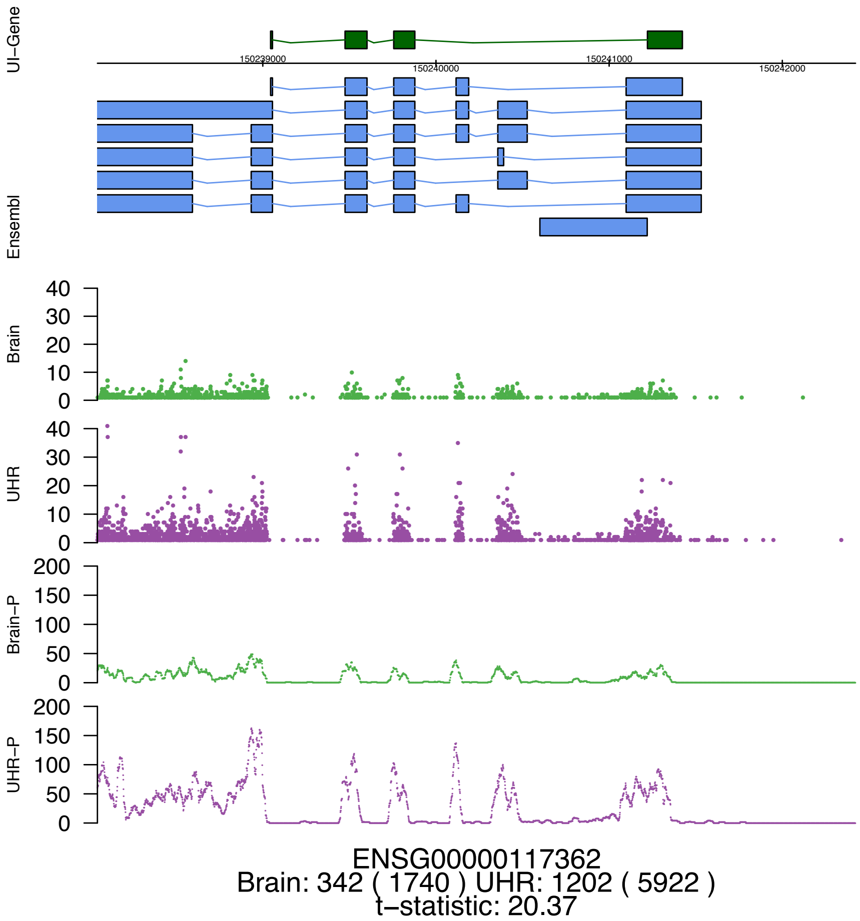

Figure SS4: *Base-level read counts.* The plot provides two representations of base-level read counts summed across the seven Brain and seven UHR lanes for Ensembl Gene ENSG00000117362. Regions corresponding to the union-intersection gene model and Ensembl transcripts are indicated by dark green and light blue boxes, respectively. The top two read tracks (Brain, UHR) display numbers of reads with 5'-end at a given base (Section S2). UI gene counts for the Brain and UHR samples are reported below the tracks; Ensembl gene counts are in parentheses. The t-statistics for UHR vs. Brain differential expression are based on GLM adjusting for flow-cell effects (1+bio+fc, Table S4). The second set of tracks (Brain-P, UHR-P) correspond to a "pileup" representation of "overlap" counts, i.e., of numbers of reads overlapping a given base.

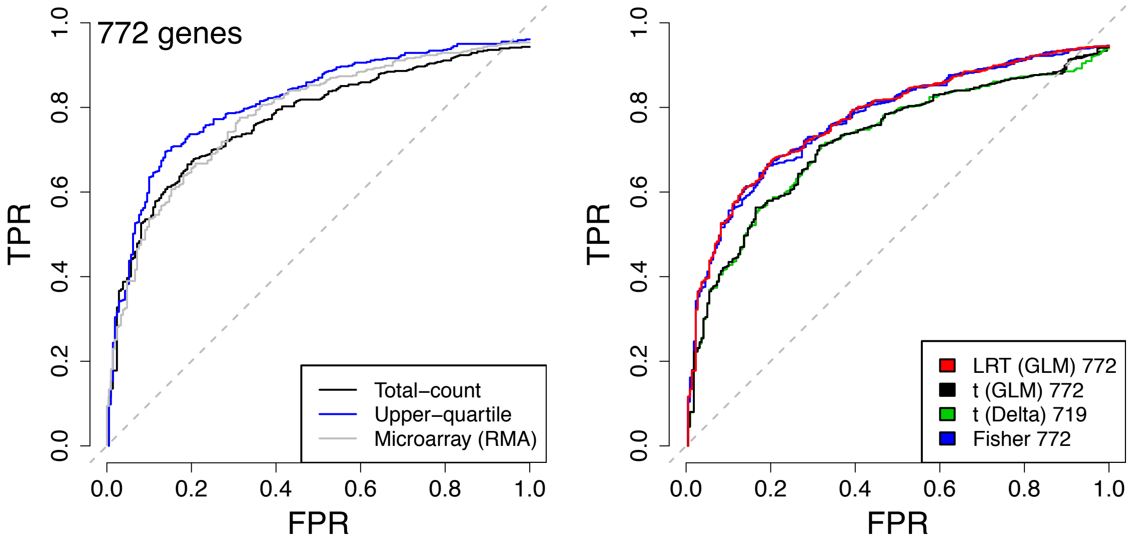

(a) Offset comparison

(b) Test statistic comparison

Figure SS5: *Comparison of normalization and test statistics: p-value based ROC curves.* ROC curves comparing the performance of, (a) normalization procedure, and (b) test statistics, computed by declaring genes DE using a .05 cutoff on the Bonferroni adjusted p-values, rather than log-fold-change. As before, in addition to satistfying the p-value cutoff, to be declared a true-positive a gene must agree in the sign of differential expression as described in Table 1.

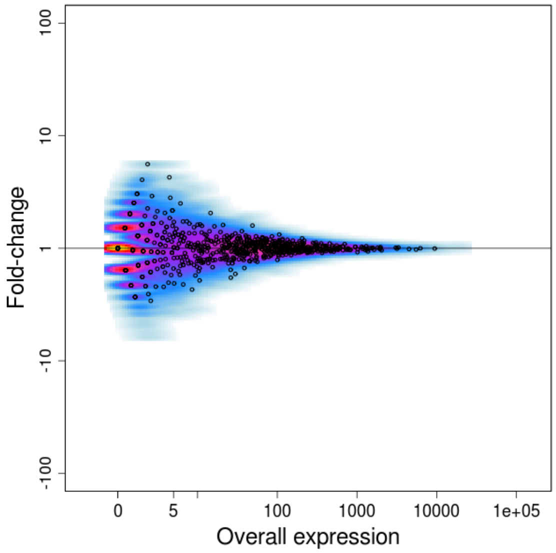

(a) MAQC-2: Replicate lanes

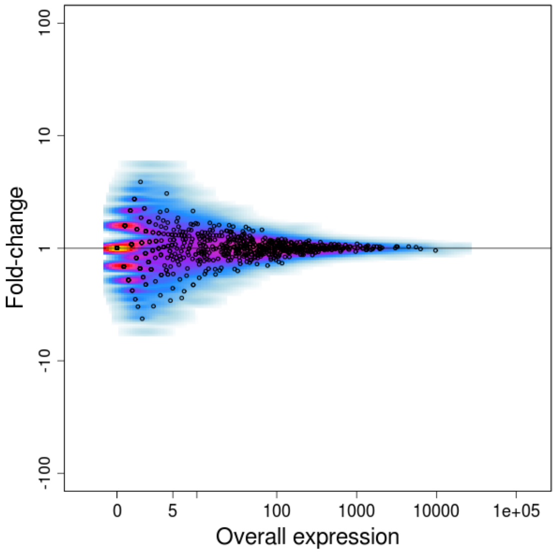

(b) MAQC-2: Lanes across flow-cells

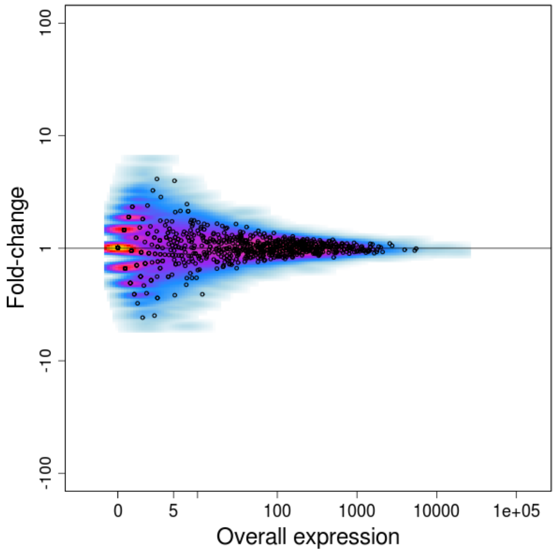

(c) MAQC-3: Lanes across library prep.

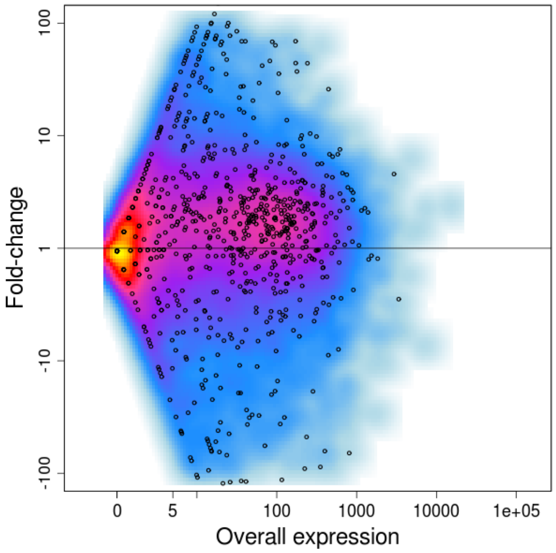

(d) MAQC-2: Lanes across biological groups

Figure SS6: *Mean-difference scatterplots of read counts across lanes, flow-cells, library preparations, and biological groups.* Scatterplots of expression fold-changes vs. overall expression measures for pairs of lanes representing different combinations of biological samples, library preparations, and flow-cells. Panel (a): Replicate Brain lanes in flow-cell F3. Panel (b): Brain lanes in flow-cell F3 vs. F2. Panel (c): UHR library preparation S4 vs. S3 lanes in flow-cell F4. Panel (d): UHR vs. Brain lanes in flow-cell F2. Only the qRT-PCR genes are individually plotted as a representative sample of genes; for comparison, these genes are plotted over the bivariate Gaussian kernel density smoothers of the MD-plots for all UI genes that contain reads in any lane of either the MAQC-2 or MAQC-3 datasets. Expression measures were normalized by total lane counts and then multiplied by 10^6 to make the scales commensurate when comparing different numbers of lanes.

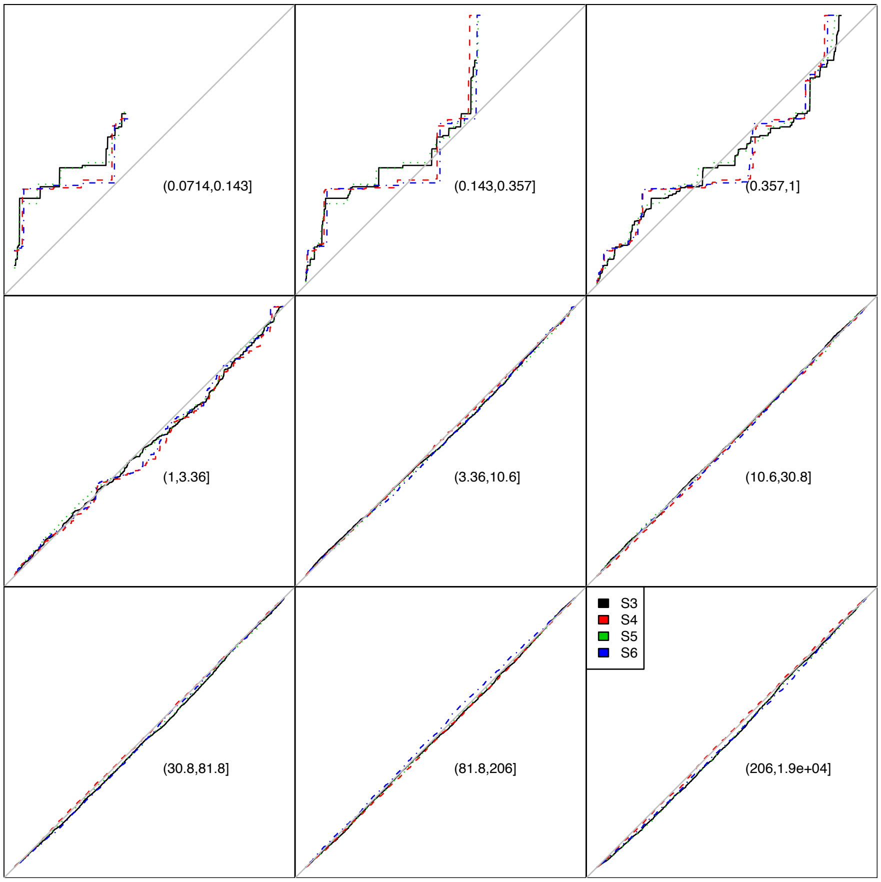

Figure SS7: *MAQC-3: Goodness-of-fit of ROI-level Poisson model for replicate lanes, by count.* The multiplicative Poisson model of Equation (1) is fit to each UI gene within library preparation. Goodness-of-fit statistics are computed and displayed in uniform quantile-quantile plots for the corresponding nominal χ^2 p-values. The QQ-plots are stratified according to UI gene counts averaged over all fourteen lanes. The count strata partition the UI genes into nine groups of approximately the same cardinality, but vastly different count ranges.

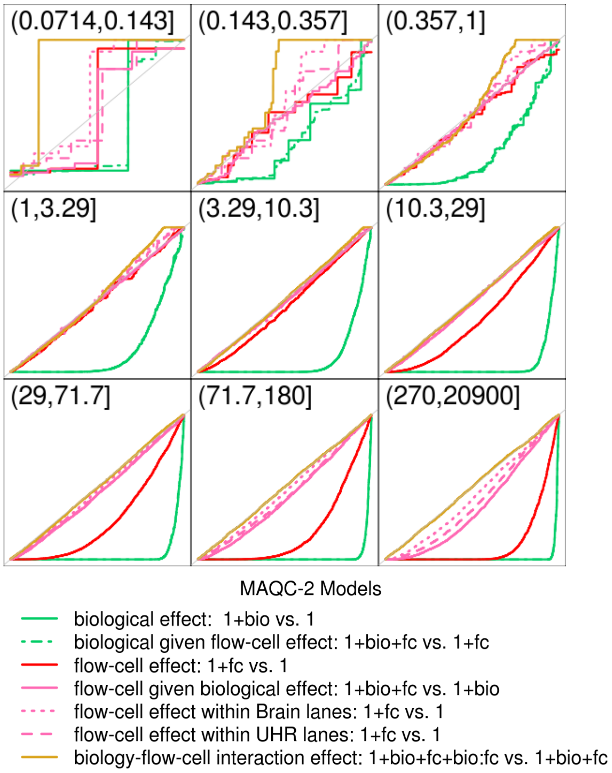

(a) MAQC-2

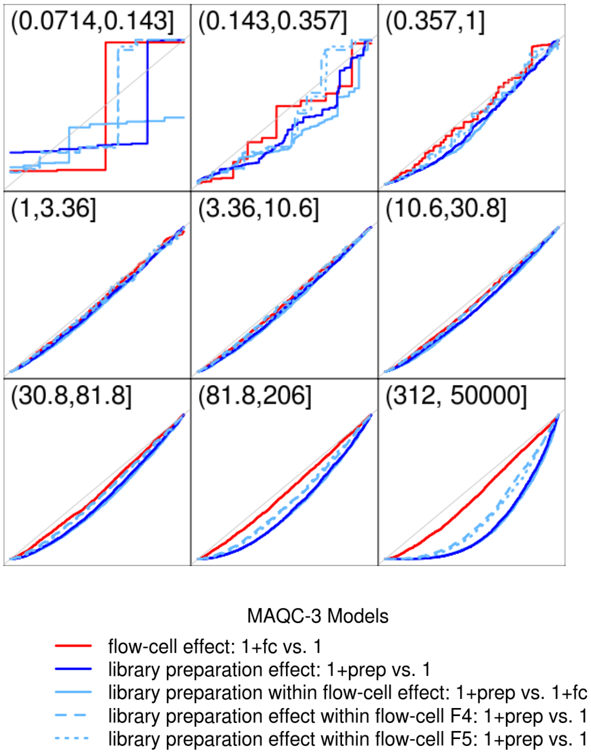

(b) MAQC-3

Figure SS8: *Count-stratified QQ-plots comparing the fit of log-linear regression models with various formulations of the biological and experimental effect parameters.* The log-linear regression model of Equation (1) is fit to each UI gene for various formulations of the biological and experimental effect parameters, λ and θ, respectively (Table S4). Models are compared with log-likelihood ratio statistics and the associated nominal χ^2 p-values are displayed in uniform quantile-quantile plots. The QQ-plots are stratified according to UI gene counts averaged over all fourteen lanes. The count strata partition the UI genes into nine groups of approximately the same cardinality, but vastly different count ranges.

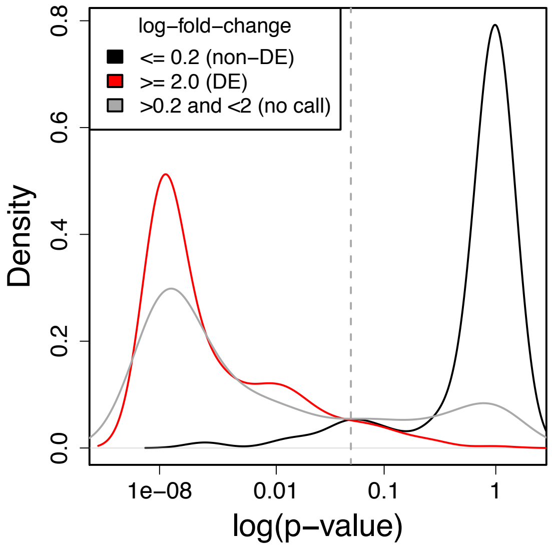

Figure SS9: *Distribution of qRT-PCR p-values for genes called DE and non-DE.* Density plots of the bonferroni-corrected p-values from qRT-PCR data, with genes separated by whether the gene was called DE, non-DE or neither ('no call').

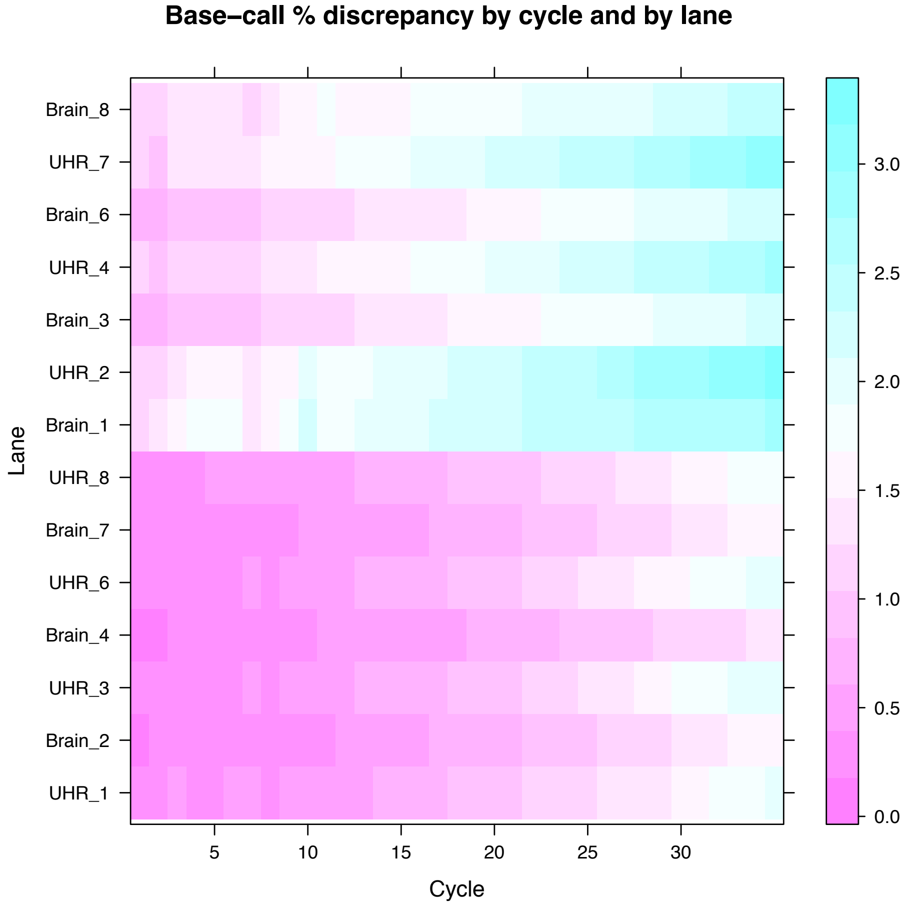

Figure SS10: *MAQC-2: Impact of phi X calibration, base-calling.* Pseudo-color image of the per cycle and per lane percentage (out of 11,244,980–13,680,634 clusters per lane) of base-calls that differ between phi X calibration and auto-calibration.

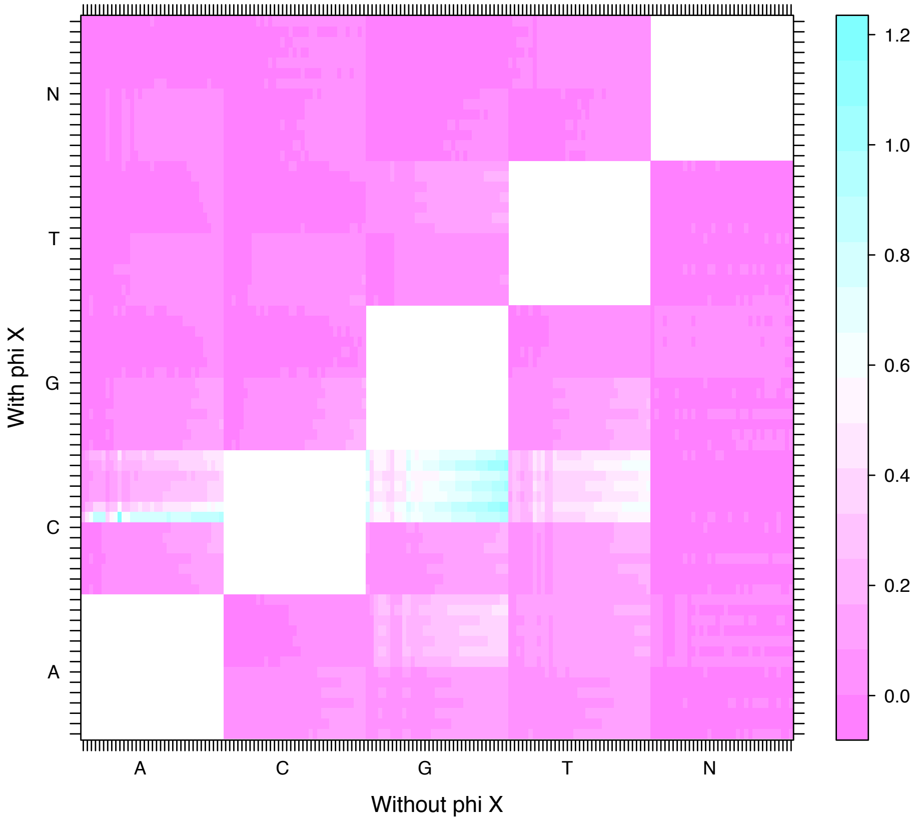

Figure SS11: *MAQC-2: Impact of phi X calibration, base-calling.* Pseudo-color image of the per cycle and per lane joint distribution of base-calls with and without phi X calibration. Each cell in the image corresponds to the percentage (out of 11,244,980–13,680,634 clusters per lane) of base-call pairs of a given type, at a given cycle and in a given lane, e.g., an (A,C) pair corresponds to a base-call of 'A' with phi X and 'C' without phi X calibration. A base-call of 'N' is returned when all four fluorescence intensities are zero. Concordant base-calls are not displayed, as they dwarf discrepant calls.

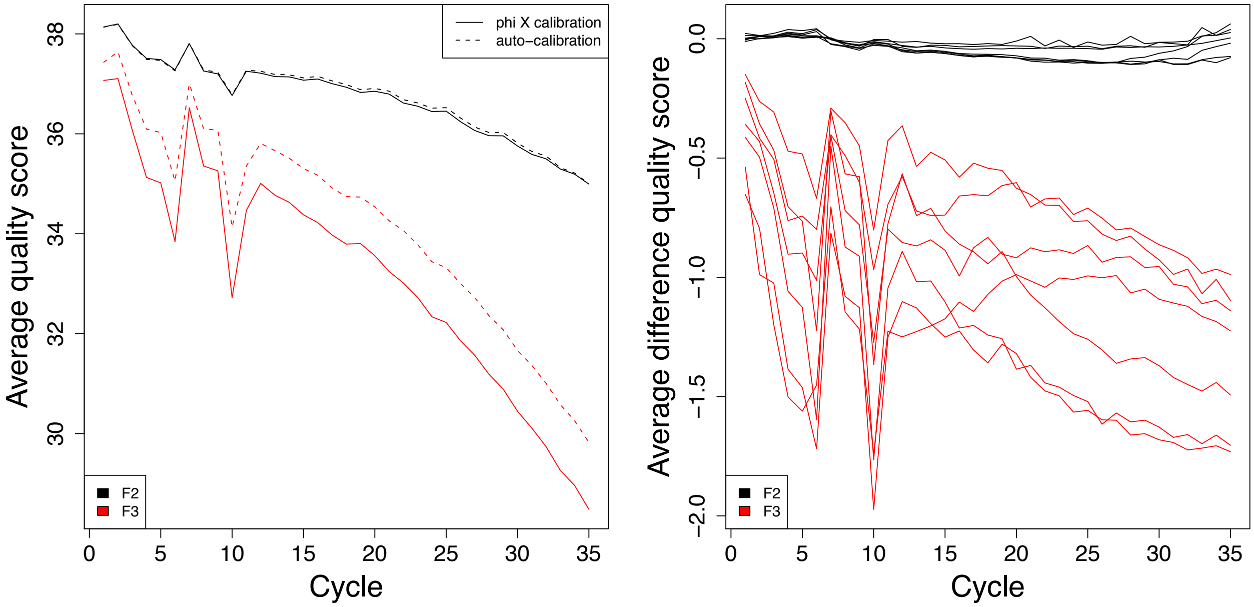

(a) All lanes combined

(b) By lane

Figure SS12: *MAQC-2: Impact of phi X calibration, quality-scoring.* Plots of per-cycle average quality scores (out of 11,244,980–13,680,634 clusters per lane) with and without phi X calibration. Panel (a): Average quality scores are averaged across seven lanes for flow-cells F2 and F3. Panel (b): Average difference of quality scores between phi X calibration and auto-calibration for fourteen lanes.

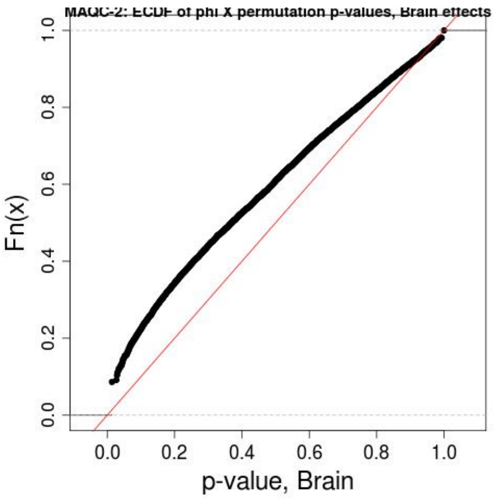

(a) Brain: p-values

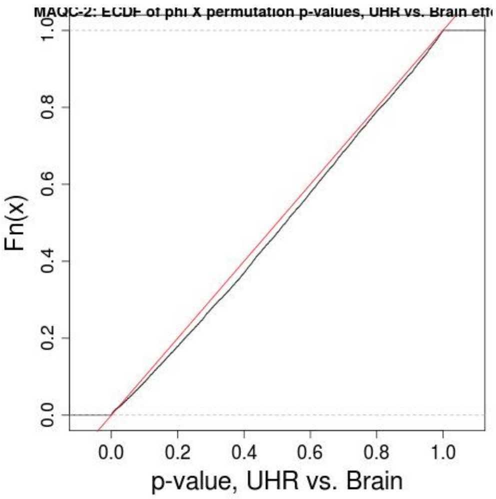

(b) UHR vs. Brain: p-values

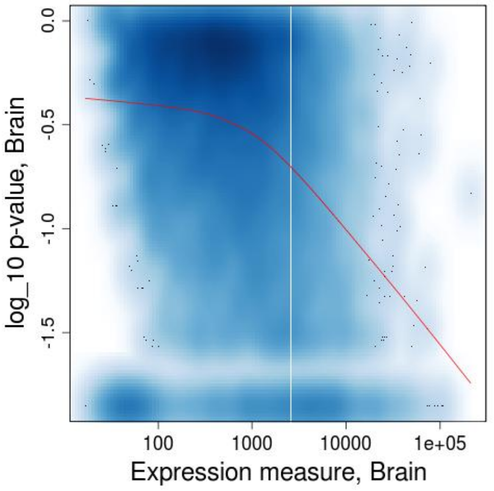

(c) Brain: p-values vs. counts

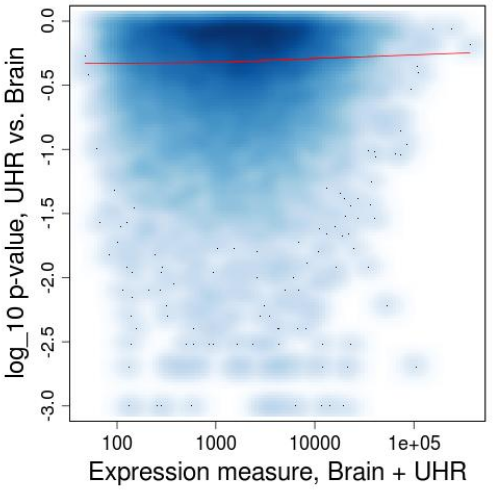

(d) UHR vs. Brain: p-values vs. counts

Figure SS13: *MAQC-2: Impact of phi X calibration, biological effect estimation.* Panel (a): Empirical cumulative function of permutation p-values for differences in Brain effects $\hat{\lambda}_{Brain,j}$ without vs. with phi X calibration. Panel (b): Empirical cumulative distribution function of permutation p-values for differences in biology effects $\hat{\lambda}_{UHR,j} - \hat{\lambda}_{Brain,j}$, i.e., expression log-ratios, without vs. with phi X calibration. Panel (c) : Bivariate binned Gaussian kernel density smoother of permutation p-values for differences in Brain effects $\hat{\lambda}_{Brain,j}$ vs. read counts summed over the seven Brain lanes. Panel (d): Bivariate binned Gaussian kernel density smoother of permutation p-values for differences in biology effects $\hat{\lambda}_{UHR,j} - \hat{\lambda}_{Brain,j}$ vs. read counts summed over all fourteen lanes. Estimates of (absolute and relative) biological effects are based on GLM with only biological effects: $\hat{\lambda}_{a,j} = \log(X_{+a,j}/X_{+a,\cdot})$, $a \in \{Brain, UHR\}$, for the UI genes having non-zero counts with both types of calibration for each of the fourteen lanes. Two-sided p-values are computed based on $1,000$ random permutations of the phi X and non-phi X sets of read counts for each of the fourteen lanes (from the possible $2^{14} = 16,384$), with a floor of $2/1,000$.

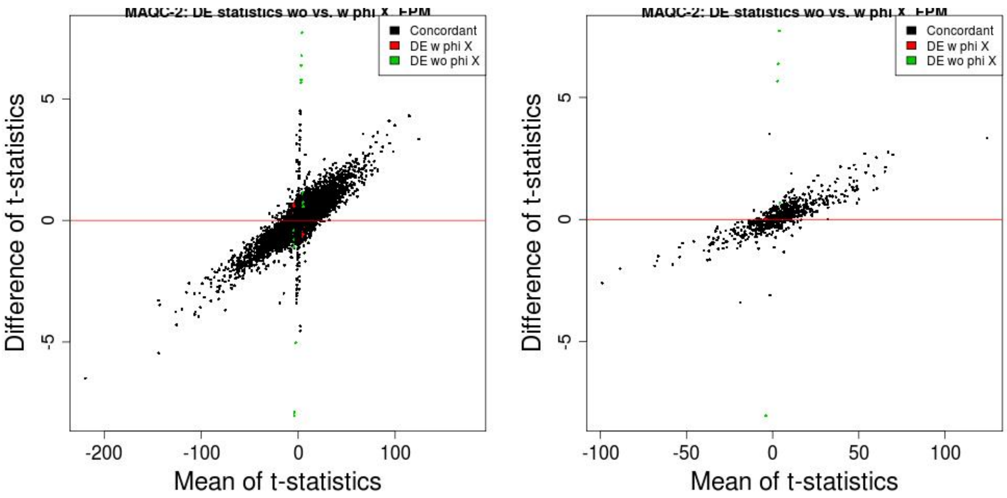

(a) All UI genes

(b) qRT-PCR genes

Figure SS14: *MAQC-2: Impact of phi X calibration, differential expression statistics – Purity-filtered perfectly matching reads (FPM).* Mean-difference scatterplots of DE statistics without vs. with phi X calibration. Panel (a): All UI genes. Panel (b): genes assayed by qRT-PCR. DE statistics are t-statistics for differences of biological effects $\lambda_{UHR,j} - \lambda_{Brain,j}$, based on GLM adjusting for flow-cell effects (1+bio+fc, Equations (1). Genes are declared differentially expressed if their nominal Bonferroni Gaussian adjusted p-values do not exceed 0.05. Discrepant DE calls are highlighted using red and green plotting symbols: red for DE according to phi X base-called lanes only and green for DE according to non-phi X base-called lanes only.
